# Deciphering molecular pathways driving cancer invasion and metastasis: advances and therapeutic prospects

**DOI:** 10.3389/fonc.2025.1684896

**Published:** 2025-12-02

**Authors:** Aziza Alshahrani, Arwa Alsubait, Zahrah Asiri, Sahar Alghamdi, Sarah Bin Saqyah, Tariq Alqahtani, Rawan Fitaihi, Njoud Altuwaijri, Yahya F. Jamous

**Affiliations:** 1 Department of Pharmacology, College of Pharmacy, King Khalid University, Abha, Saudi Arabia; 2 Medical Research Core Facility and Platforms Department, King Abdullah International Medical Research Center (KAIMRC), Ministry of National Guard Health Affairs, Riyadh, Saudi Arabia; 3 Department of Clinical Laboratory Sciences, College of Applied Medical Sciences, King Saud Bin Abdulaziz University for Health Sciences, Riyadh, Saudi Arabia; 4 Department of Pharmaceutics, College of Pharmacy, King Khalid University, Abha, Saudi Arabia; 5 Department of Pharmaceutical Sciences, College of Pharmacy, King Saud Bin Abdulaziz University for Health Sciences, Riyadh, Saudi Arabia; 6 King Abdulaziz Medical City, National Guard Health Affairs (NGHA), Riyadh, Saudi Arabia; 7 Department of Preclinical Studies, Benefit and Risk Assessment Executive Directorate, Drug Sector – Saudi Food and Drug Authority, Riyadh, Saudi Arabia; 8 Department of Pharmaceutics, College of Pharmacy, King Saud University, Riyadh, Saudi Arabia; 9 Wellness and Preventative Medicine Institute, Health Sector, King Abdulaziz City for Science and Technology (KACST), Riyadh, Saudi Arabia

**Keywords:** cancer metastasis, mesenchymal-epithelial transition, pre-metastatic niche, tumor heterogeneity, liquid biopsy, therapeutic resistance

## Abstract

Metastasis is the primary cause of cancer-related mortality worldwide. This narrative review integrates recent advances in the molecular circuits orchestrating metastatic progression, encompassing epithelial–mesenchymal transition (EMT), organotropism, extracellular matrix remodeling, angiogenesis, hypoxia-inducible signaling, tumor-cell migration modes, and tumor–immune interactions through expert-guided literature selection. We examined therapeutic innovations that disrupt these pathways, including EMT modulators, matrix metalloproteinase inhibitors, VEGF/VEGFR-targeted regimens, hypoxia-activated prodrugs, and next-generation immunotherapies such as immune checkpoint blockade and chimeric antigen receptor T cells. Additionally, we discuss established nanotechnology-based delivery systems, advancing multi-omics integration, evolving single-cell analyses, and emerging CRISPR-Cas9 gene-editing applications as tools for improving metastasis detection, monitoring, and treatment. Despite this progress, translational obstacles persist, particularly regarding intratumoral heterogeneity, adaptive resistance, and limited preclinical model fidelity. Addressing these challenges requires biomarker-guided, multi-target therapeutic combinations, interdisciplinary collaboration, and globally inclusive clinical trials. This evidence underscores the importance of integrated strategies that simultaneously target intrinsic tumor plasticity and microenvironmental support to transform metastatic cancer outcomes.

## 1 Introduction

**Figure 1:**
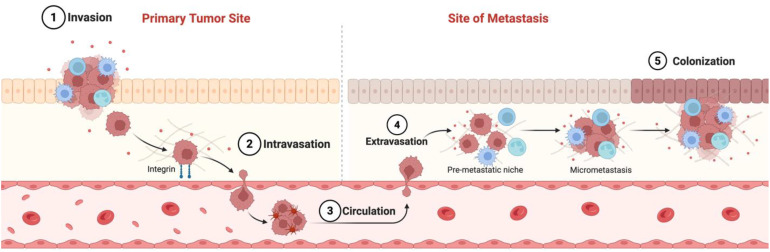
The metastatic cascade refers to the sequence of events in which aggressive cancer cells detach from the original tumor, circulate through the bloodstream, and ultimately arrive at distant organs, where they can form one or more metastases. Created with BioRender.com.

Cancer metastasis is the process by which malignant tumor cells spread from their original sites to distant organs, resulting in secondary tumors. It accounts for approximately 90% of cancer-related deaths, rendering it a critical area of research and clinical focus (1). This complex, multi-step process, known as the metastatic cascade (**Figure 1**), involves local invasion, intravasation into the circulation, survival of circulating tumor cells (CTCs), extravasation, and colonization at secondary sites. Each step is intricately linked to various cellular and molecular mechanisms that dictate the behavior of cancer cells and their interactions with the surrounding environment (2). The invasion process begins when tumor cells lose their adhesion to neighboring cells, allowing them to break away from the primary tumor and invade surrounding tissues. This is accomplished through the degradation of the basement membrane and extracellular matrix (ECM), along with the regulation of proteins that control cell movement (3). After invasion, tumor cells undergo intravasation, penetrating the lymphatic or blood circulatory systems, and subsequently extravasation, where they enter new tissues to form secondary tumors (4). The ability of these cells to survive in the bloodstream, adapt to foreign microenvironments, and establish new growth at distant sites is influenced by multiple factors, including ECM composition and stiffness, which can promote or inhibit tumor progression (5). Moreover, the tumor microenvironment (TME) plays a significant role in metastasis. Cancer-associated fibroblasts (CAFs), immune cells, and various growth factors interact with tumor cells, thereby altering their invasive capabilities and facilitating metastasis (6). For instance, CAFs can remodel the ECM, making it more permissive for tumor invasion and promoting further cancer progression through cytokine and matrix-degrading enzyme release (7). All these factors influence metastatic potential and therapy resistance.

Understanding these pathways is pivotal for identifying novel therapeutic targets and underpins emerging precision medicine strategies (8, 9). For example, matrix metalloproteinases (MMPs) are central regulators of cell behavior and ECM remodeling, and their dysregulation facilitates invasion and metastasis (10). Advances in genomic and multi-omics profiling have clarified that metastasis is not a uniform process; distinct tumors and even separate metastatic lesions exhibit heterogeneous molecular signatures and unique organotropism patterns (11, 12). Consequently, personalized approaches that account for this diversity are increasingly necessary.

**Figure 2:**
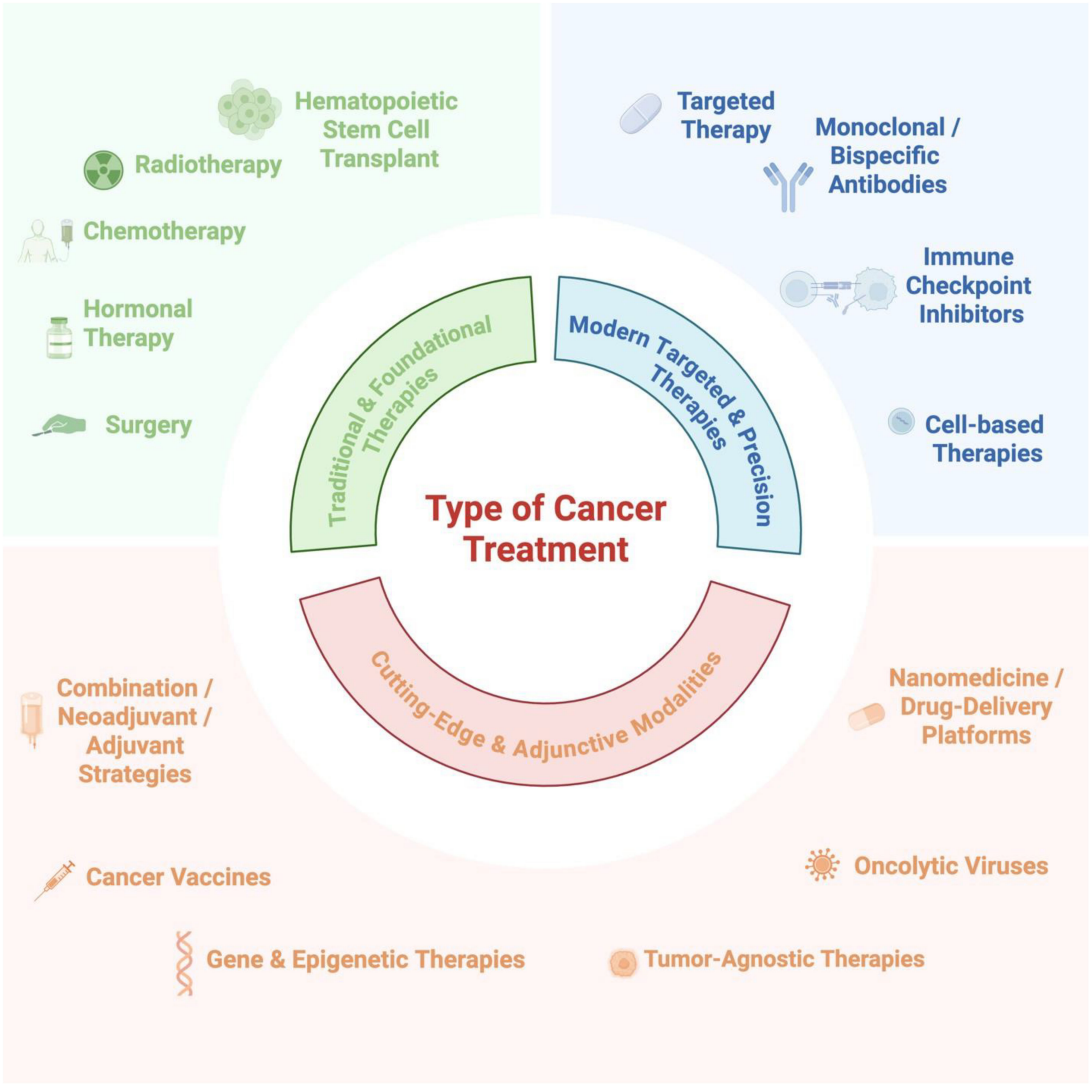
Available treatment options consist of traditional and foundational therapies, modern targeted and precision therapies, and cutting-edge and adjunctive modalities, among others. Created with BioRender.com.

Traditional cancer therapies, including surgery, radiotherapy, chemotherapy, hormone therapy, and immunotherapy (**Figure 2**), have historically prioritized the reduction of the primary tumor bulk, often with limited effects on established or micrometastatic disease (13). while newer anti-cancer drugs, such as neutralizing antibodies and small-molecule inhibitors, often address metastasis only as a secondary effect. Newer classes of agents, such as migrastatics that target the migratory and invasive capacity of cancer cells, are promising but still require clinical validation (14). Integrating antimetastatic strategies with precise biomarkers detected through liquid biopsy and integrated multi-omics technologies is essential for real-time disease monitoring, individualized treatment, and improved outcomes (15, 16).

Despite these advances, the field faces persistent challenges. Tumor heterogeneity complicates the identification of universal treatment regimens, as genetic, epigenetic, and microenvironmental factors result in variable therapeutic responses, resistance, and disease progression (13, 17–19). Furthermore, several antimetastatic therapies have failed in clinical trials due to inadequate efficacy or unanticipated toxicity (20, 21), often reflecting an incomplete understanding of metastatic biology and the influence of factors such as ECM composition, hypoxia, angiogenesis, and the immune system. Addressing these multifaceted obstacles requires multi-targeted therapeutic regimens that disrupt both the tumor-intrinsic and microenvironmental drivers of metastasis, supported by continued innovation in diagnostics and drug development. In this review, we critically examine the molecular and cellular mechanisms underlying cancer invasion and metastatic progression and discuss recent and emerging therapeutic strategies targeting these pathways.

## 2 Key molecular pathways involved in cancer invasion and metastasis

### 2.1 Epithelial–mesenchymal transition

**Figure 3:**
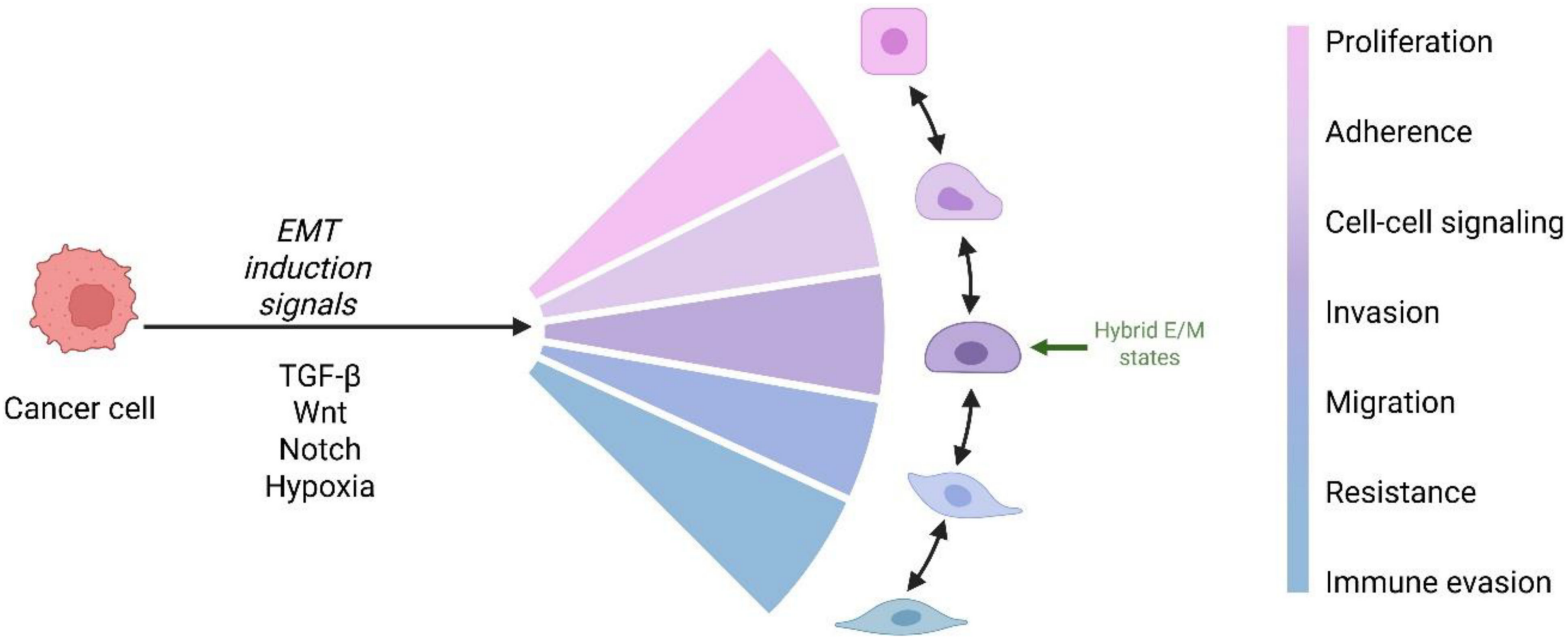
Epithelial–mesenchymal plasticity (EMP) in cancer. Signals such as TGF-β, Wnt, Notch, and hypoxia induce EMT, generating hybrid epithelial/mesenchymal (E/M) states with mixed traits. These states enhance adaptability through invasion, migration, resistance, and immune evasion, while MET at distant sites supports metastatic colonization. Adapted from Kuburich et al. (2023), *Semin Cancer Biol*. Created with BioRender.com.

Epithelial–mesenchymal transition (EMT) is a cellular mechanism by which epithelial cells lose specific epithelial features and gain mesenchymal traits, thereby increasing cell motility. EMT activation is essential in physiological settings for numerous embryonic developmental processes, such as gastrulation, neural crest development, cardiac valve formation, and wound healing (22). However, under pathological conditions, EMT is a pivotal contributor to organ fibrosis and is a critical process enabling cancer progression and metastasis, allowing epithelial tumor cells to disseminate from the primary site to distant organs (22–27). However, histological analyses of human carcinoma metastases have revealed that most distant lesions retain epithelial characteristics similar to the primary tumor, making it difficult to distinguish EMT-derived tumor cells from stromal cells (28–30). These observations fueled debate, particularly among pathologists, over whether EMT truly contributes to human metastasis. However, experimental models demonstrate that EMT activation enables dissemination, whereas mesenchymal–epithelial transition (MET) at distant sites facilitates colonization and metastatic outgrowth (29–35). Research has shown that EMT is not a binary process but generates intermediate hybrid epithelial–mesenchymal (E/M) states, many of which do not progress to a fully mesenchymal phenotype (36, 37). While EMT confers invasive and migratory properties fundamental to embryonic development and cancer progression, in pathological contexts such as organ fibrosis, the program remains partial, producing hybrid E/M cells with limited migratory activity (38, 39). In cancer, however, hybrid E/M cells can be highly invasive and possess strong metastatic potential (40–42). These intermediate states are part of a broader concept termed epithelial–mesenchymal plasticity (EMP), which allows cancer cells to reversibly occupy position along the E/M spectrum. As illustrated in **Figure 3**, this plasticity enables dynamics switching between epithelial, mesenchymal, and hybrid phenotypes, conferring trait such as invasion, migration, immune evasion, and resistance. These intermediate states, together with EMT’s transient and plastic nature, underscore the nonlinear dynamics of this process. The ability of cancer cells to transition between epithelial, mesenchymal, and hybrid states is regulated by transcription factors, signaling pathways, and epigenetic mechanisms (43). A study using an inducible mouse model of squamous cell carcinoma (SCC) found that switching on TWIST1 triggers EMT, aiding cancer cells to break away and enter the bloodstream. However, for these cells to settle in new organs and grow into large metastases, TWIST1 must be downregulated to stop the EMT process (33). Tran et al. reported similar findings in an inducible Snail1 mouse model of breast cancer (44). Collectively, these studies demonstrated that while TWIST1- or SNAI1-driven EMT is highly effective in promoting tumor invasion, bloodstream entry (intravasation), and exit at distant sites (extravasation), the reversal of MET is essential for the growth of metastases in distant organs. Reichert et al. found that the loss of p120-catenin (p120ctn) in a mouse model is sufficient to trigger EMT and enhance metastasis development. Interestingly, the loss of both p120ctn and E-cadherin appears to drive metastasis, specifically to the lungs, whereas their expression is necessary to enable MET and support metastasis in the liver (45). This indicates that EMT may play a role in determining the organ-specific spread of metastasis. Complementing EMT-promoting transcription factors, several metastasis suppressor genes (MSGs), such as CADM1, BRMS1, and KAI1/CD82, function to restrict metastatic dissemination without affecting primary tumor growth. For example, CADM1 enhances immune-mediated clearance of tumor cells and suppresses tumor invasiveness by modulating cell–cell adhesion and natural killer (NK) cell-mediated cytotoxicity (46). BRMS1 impedes metastatic spread by repressing EMT-associated transcriptional networks, including NF-κB and Twist signaling, and by modulating chromatin remodeling complexes (47). KAI1/CD82, a member of the tetraspanin family, maintains epithelial adhesion by stabilizing E-cadherin at the plasma membrane and inhibiting cell motility and invasion (48). Loss or downregulation of these MSGs is frequently observed in aggressive tumors and correlates with poorer clinical outcomes, underscoring their critical roles as intrinsic molecular brakes on the metastatic cascade. In addition to EMT–MET dynamics, metastatic dormancy represents a critical barrier to long-term cancer control (49). Disseminated tumor cells (DTCs) can enter a quiescent state, often remaining undetectable for years before reactivating to form overt metastases (50). This dormant state is regulated by molecular signals such as p38 MAPK, TGF-β2, and BMP, as well as niche-derived cues that suppress proliferation and modulate immune evasion (49). Reactivation from dormancy—often triggered by inflammation or niche remodeling—is clinically associated with late relapse, particularly in breast and prostate cancers (50).

### 2.2 Organotropism

Different cancer types exhibit organ-specific metastasis patterns. Gastric, gallbladder, pancreatic, and colorectal cancers typically develop liver metastases before colonizing distant organs, such as the lungs. Breast cancer cells frequently disseminate to preferred metastatic sites, including the lungs, bone, brain, and liver, whereas prostate cancer cells primarily spread to bone (5, 51). The distribution of CTCs throughout the body is influenced by blood flow patterns. As the liver and lungs are among the first organs that CTCs reach, they frequently serve as primary sites of metastasis in many cancer types. Additionally, gaps in the liver and bone marrow sinusoids facilitate the extravasation of CTCs, contributing to a higher occurrence of metastases in these organs (52). Although anatomical characteristics contribute to the preferential spread of cancer to specific organs, they do not fully explain the nonrandom patterns observed in metastasis. The key mechanisms that drive organotropism include chemokine signaling, vascular niche formation, and immune interactions.

#### 2.2.1 Chemokine signaling

Many studies have highlighted the significant role of chemokine signaling in organ-specific metastasis. The CCR7/CCL21 axis has been shown to drive lymph node metastasis in several cancers (53). Research has indicated that CCR7 expression correlates with increased lymphatic spread in esophageal SCC, reinforcing its role in tumor progression (54). Similarly, in gastric cancer, high CCR7 expression has been linked to metastasis to the regional lymph nodes, suggesting that targeting this pathway may be a potential strategy to limit disease dissemination (55). Another well-characterized chemokine axis, the CXCL12/CXCR4 axis, plays a critical role in guiding cancer cells to the bone marrow, lungs, and liver, which are common metastatic sites for various tumors. Studies indicate that CXCL12 is highly expressed in these organs, creating a chemotactic gradient that attracts CXCR4-expressing tumor cells, thereby facilitating metastasis (56). These findings reinforce the importance of chemokine-driven organotropism in cancer metastasis and suggest that targeting these signaling pathways may provide novel therapeutic strategies for the prevention and management of metastatic diseases.

#### 2.2.2 Vascular niche formation

The formation of pre-metastatic niches (PMNs) significantly influences organotropism during cancer metastasis. Primary tumor-release factors prepare distant organs for future metastasis by altering the local microenvironment. These alterations include ECM remodeling, increased vascular permeability, and recruitment of bone marrow-derived cells, creating a supportive niche for incoming tumor cells (57). For instance, cancer-derived exosomal miR-25-3p promotes PMN formation by inducing vascular permeability and angiogenesis (58). These pre-metastatic niches determine the organ-specific spread of metastasis, thereby influencing cancer cell organotropism (59). Vascular niche formation explains why cancer spreads to specific organs. This knowledge may result in the development of novel therapies that block supportive environments.

#### 2.2.3 Immune interactions

Immune interactions play a pivotal role in organotropism and in the tendency of cancer cells to metastasize to specific organs. The dynamic interplay between tumor cells and the host immune system significantly influences the establishment and progression of metastasis. Emerging evidence suggests that tumor cells can recruit immune cells, which, in turn, promote tumor cell invasion, survival in circulation, and colonization of different organs (60). Additionally, the formation of a PMN, a microenvironment conducive to tumor cell colonization, is influenced by immune components. Primary tumors can modulate distant sites by recruiting bone marrow-derived cells and altering the local immune environment, thereby promoting organ-specific metastasis (61). Understanding these immune interactions is crucial for the development of therapeutic strategies aimed at modulating the immune system to prevent or treat organ-specific metastases.

Building upon the principles of organ-specific metastasis, the role of cell adhesion molecules (CAMs) is pivotal in modulating both tumor cell detachment and intravasation and homing and colonization at distant sites.

### 2.3 Cell adhesion molecules and ECM remodeling in cancer metastasis

CAMs including cadherins, integrins, and selectins are crucial for tissue architecture and metastatic dissemination (62, 63). In epithelial cancers, loss of E-cadherin (the “cadherin switch” to N-cadherin) during EMT disrupts adherens junctions and enhances cell motility and invasiveness (62). Aberrant integrin expression (e.g., αvβ3 and α5β1) strengthens tumor cell attachment to the ECM and activates FAK/Src–PI3K/AKT signaling, driving migration through tissue barriers (63). In the bloodstream, selectins (E-, P-, and L-selectin) mediate rolling adhesion of CTCs on activated endothelium, a process analogous to leukocyte extravasation, thereby aiding tumor cell arrest and extravasation at distant sites (64). Dysregulation of these CAMs fuels metastasis: for example, reduced E-cadherin correlates with higher invasiveness and poor prognosis, while overactive integrins enhance survival and migration (62, 63). Integrin-dependent CAM–ECM interactions link CAM biology with dynamic ECM remodeling, which is pivotal for metastasis. Tumor-associated ECM undergoes extensive remodeling to facilitate invasion. Proteolytic enzymes such as MMPs are major drivers: MMP-2 and MMP-9 (gelatinases) degrade basement membrane type IV collagen and interstitial collagens, clearing paths for invading cells (65, 66). Elevated MMP-9 levels, for instance, are clinically associated with lymph node metastasis and poor survival in breast cancer. ECM degradation not only removes physical barriers but also releases bound growth factors and bioactive matrikines that promote angiogenesis and further tumor growth (65, 66). Concurrently, enzymes like lysyl oxidase (LOX) crosslink collagen and elastin, increasing matrix stiffness. CAF- and tumor-derived TGF-β induces LOX expression in CAFs, leading to collagen crosslinking and a rigid ECM that paradoxically supports tumor cell migration and formation of PMN (66).

Aberrant ECM remodeling can also create a dense, fibrotic desmoplastic stroma that impedes treatment. In pancreatic and other tumors, massive collagen and hyaluronan deposition raise interstitial pressure and block drug delivery (67). A stiff matrix amplifies integrin signaling in cancer cells, further enhancing proliferation and survival. Non-malignant stromal cells amplify these effects: CAFs are key architects of the remodeled ECM. CAFs deposit new matrix proteins (e.g., collagens and fibronectin) and secrete growth factors (TGF-β, VEGF, PDGF, FGF2, and HGF) and cytokines (IL-6, IL-8, and CXCL12/SDF-1) that jointly reprogram the matrix and tumor behavior (66). For example, CAF-derived TGF-β activates LOX and fibronectin assembly, reinforcing a stiff, fibrillar ECM (66). CAFs also produce multiple MMPs (MMP-1, -2, and -9) to clear ECM barriers. These CAF–tumor interactions are now targeted therapeutically: agents against fibroblast activation protein (FAP) on CAFs (e.g., FAP-specific CAR-T cells or inhibitors) and inhibitors of CAF-derived signals (e.g., TGF-β or IL-6 blockade) are being explored to disrupt the tumor-supportive stroma (66, 68).

Immune cells within the TME further modulate the ECM. Tumor-associated macrophages (TAMs) secrete MMPs and other proteases that degrade ECM and promote metastasis. Importantly, a rigid, collagen-rich matrix can skew immune cell function toward an immunosuppressive phenotype (69). For instance, stiff ECM promotes accumulation of regulatory T cells and myeloid-derived suppressor cells, creating barriers to immune infiltration and therapy. Thus, the reciprocal interplay of CAMs and ECM remodeling establishes a feed-forward loop driving invasion. Targeting this axis—for example, by LOX or integrin inhibitors, MMP modulators, or drugs that normalize ECM stiffness—is a promising strategy to limit metastatic spread and improve treatment outcomes (66, 69).

### 2.4 Hypoxia, angiogenesis, and lymphangiogenesis in metastasis

**Figure 4:**
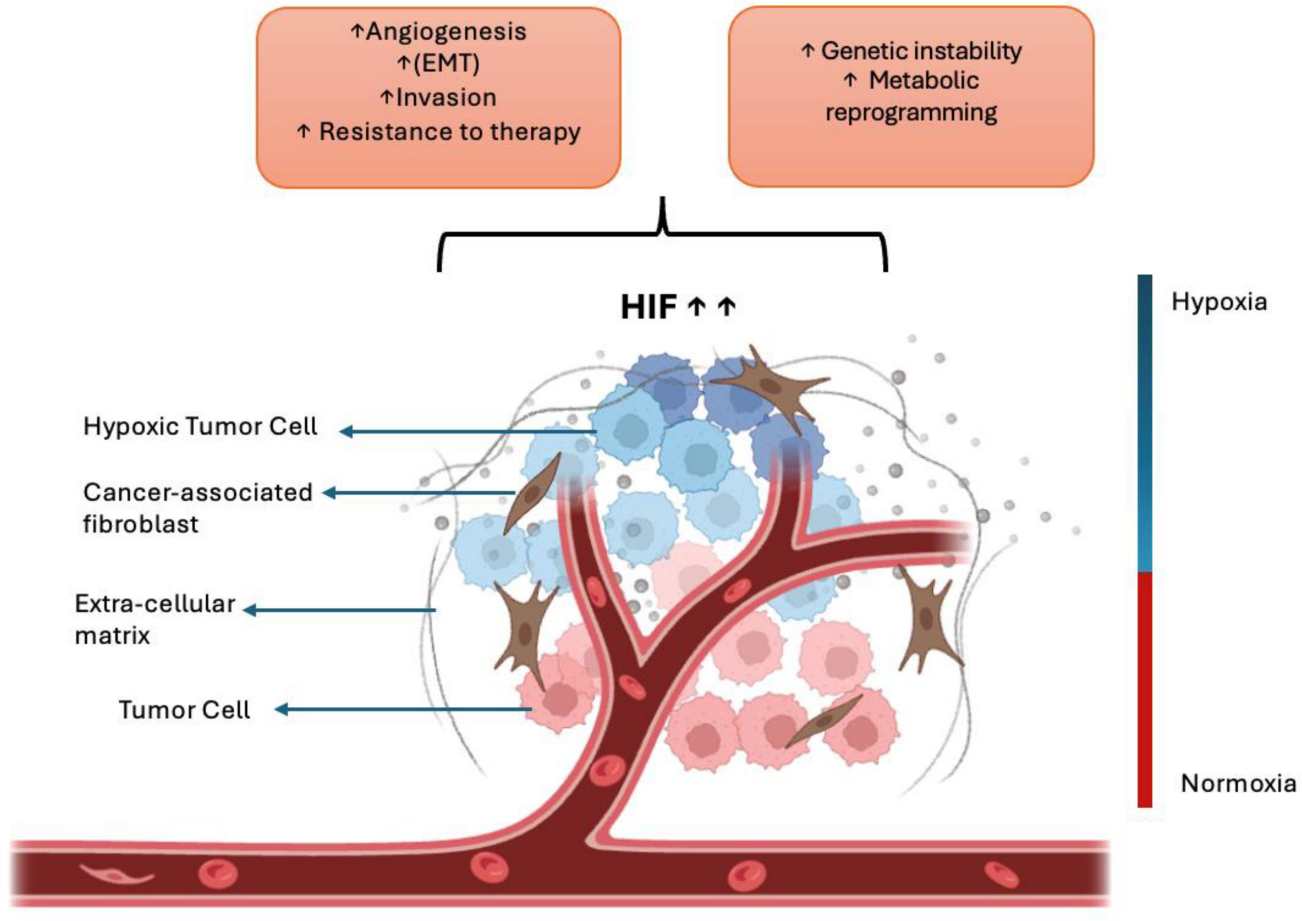
Hypoxia-driven mechanisms in cancer metastasis. Under low-oxygen conditions, cancer cells stabilize hypoxia-inducible factors (HIF-1α and HIF-2α), which activate downstream signaling pathways that promote angiogenesis, epithelial–mesenchymal transition (EMT), invasion, and therapeutic resistance. These molecular adaptations enable cancer cells to escape from the primary tumor and metastasize to distant organs. Created with BioRender.com.

Hypoxia (low oxygen) in the TME strongly drives metastasis. Under hypoxic stress, cancer cells stabilize HIF-1α and HIF-2α, which activate genes promoting angiogenesis, EMT, invasion, and therapy resistance (70, 71). These adaptations increase cell motility and survival, enabling tumor cells to escape the primary site and colonize distant organs. Hypoxia also impairs DNA repair and causes genomic instability, and it reprograms cellular metabolism to support an invasive phenotype (72). Clinically, tumors with high hypoxia or HIF levels exhibit more metastases and poorer patient outcomes (72). One key consequence of hypoxia is the induction of angiogenesis (**Figure 4**)—new blood vessels that supply the tumor with oxygen and nutrients and provide routes for dissemination (73). Angiogenesis and lymphangiogenesis are fundamental processes enabling tumor growth and spread. Angiogenesis—the sprouting of new blood vessels from existing vasculature—is driven by growth factors such as VEGF (and FGF) (73). It provides tumors with nutrients and oxygen and creates conduits for tumor cells to enter the bloodstream. Importantly, higher intratumoral microvessel density (a measure of angiogenesis) strongly correlates with increased metastatic potential and worse prognosis (73).

**Figure 5:**
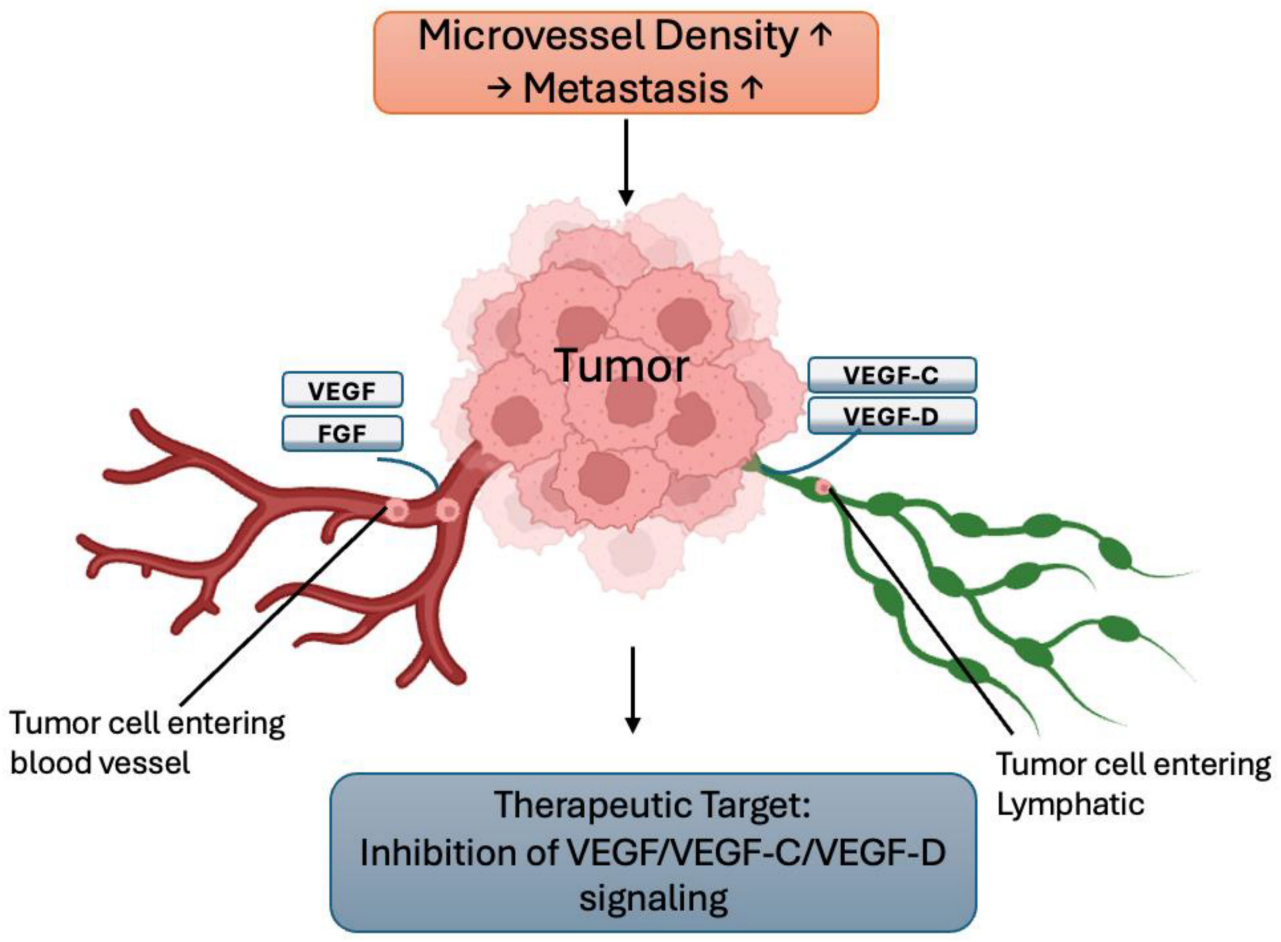
Tumor-induced angiogenesis and lymphangiogenesis in metastatic progression. Tumor-secreted growth factors (VEGF, FGF, VEGF-C, and VEGF-D) stimulate the formation of new blood vessels (angiogenesis) and lymphatic vessels (lymphangiogenesis), providing routes for cancer cell dissemination to distant organs and regional lymph nodes, respectively. Increased vascular density correlates with enhanced metastatic potential and poor prognosis. Created with BioRender.com.

Lymphangiogenesis—the formation of new lymphatic vessels—is similarly induced by factors like VEGF-C and VEGF-D (73), which facilitate tumor cell entry into lymphatic capillaries and spread to regional lymph nodes. Both angiogenesis and lymphangiogenesis thus serve as prognostic indicators and therapeutic targets: blocking VEGF/VEGFR or related signaling can limit metastatic dissemination. **Figure 5** illustrates tumor-induced angiogenesis and lymphangiogenesis under these conditions.

### 2.5 Tumor cell migration mechanisms

Tumor cell migration is a highly adaptable process involving several distinct mechanisms that enable cancer cells to invade the surrounding tissues and metastasize. These mechanisms include individual migration, such as mesenchymal and amoeboid movement, and collective migration, where groups of cells move together while maintaining cell–cell junctions (74). Mesenchymal migration is characterized by an elongated cell morphology, strong adhesion to the ECM via integrins, and proteolytic degradation of ECM components, often facilitated by MMPs (74, 75). In contrast, amoeboid migration involves rounded, highly deformable cells that can squeeze through ECM gaps with minimal proteolysis, relying more on cytoskeletal contractility and less on adhesion (74). Collective migration allows clusters or sheets of tumor cells to invade while preserving intercellular connections, providing resistance to environmental stress (75). The plasticity of tumor cells enables them to switch between these migration modes in response to microenvironmental cues, such as ECM stiffness or therapeutic interventions, which complicates efforts to target metastasis (74, 75). At the molecular level, migration is driven by actin polymerization, formation of membrane protrusions (lamellipodia and filopodia), and dynamic regulation of adhesion complexes, all tightly coordinated by signaling pathways and mechanical forces (76).

Given the reciprocal interplay between migrating tumor cells and host immunity, understanding the dualistic role of the immune system in constraining or promoting metastasis is essential.

### 2.6 Tumor–immune system interactions

Tumor–immune system interactions represent a complex and dynamic relationship that critically influences cancer progression and metastasis. Rather than passively escaping immunosurveillance, metastatic cancer cells actively hijack and reprogram the immune system to facilitate progression through a metastatic cascade (60). This bidirectional relationship evolves throughout tumor development, with immune cells initially demonstrating anti-tumor effects during the early invasion stages, before transitioning into tumor-promoting phenotypes as the disease advances (77). Tumors employ multiple immune evasion mechanisms, including restricting antigen recognition, creating immunosuppressive microenvironments, and exploiting immune checkpoints (78). Particularly important is how cancer cells undergoing EMT contribute to immune suppression, allowing them to escape from cytotoxic T cells and NK cells (60). Immune cells, particularly those of the myeloid lineage, reciprocally support metastasis by secreting inflammatory factors that promote invasion, angiogenesis, and matrix remodeling (79). These interactions extend beyond the primary tumor, as tumor–immune crosstalk generates premetastatic niches in distant organs, facilitating colonization and determining organ-specific metastatic patterns, or “organotropism” (60, 80).

Understanding these complex interactions provides critical insights for developing immunotherapeutic strategies that disrupt tumor–immune collaboration and enhance anti-tumor immunity.

## 3 Advances in therapeutic targeting of metastasis

### 3.1 Targeting EMT pathways

One therapeutic approach involves inhibiting EMT-activating transcription factors (EMT-TFs) such as Snail, Twist, and ZEB1/2 through RNA interference (RNAi)-based approaches, antisense oligonucleotides, or vaccine-based modalities (81).

A complementary therapeutic strategy involves inhibiting the upstream signaling pathways that sustain EMT, particularly TGF-β, Wnt/β-catenin, NF-κB, Notch, RAS, and PI3K/Akt. Several pharmacological and repurposed agents have shown potential to disrupt these networks. For example, the phosphodiesterase-4 inhibitor rolipram and the statin simvastatin attenuate TGF-β-induced EMT, while suramin (a heparanase inhibitor) and olaparib (a PARP inhibitor) suppress EMT in TGF-β-driven models (82–84). Collectively, these findings underscore that targeting EMT-related signaling, even with drugs of established safety profiles, can modulate metastatic behavior and may facilitate rapid clinical translation.

Increasing attention has been directed toward non-coding RNAs (ncRNAs), including microRNAs (miRNAs), long non-coding RNAs (lncRNAs), and circular RNAs (circRNAs) (85). These molecules serve as critical regulators of EMT and contribute to both intrinsic and acquired resistance to cancer therapies (86). Tumor-suppressive miRNAs, such as the miR-200 family and miR-34, have been shown to directly suppress EMT-TFs, whereas oncogenic lncRNAs and circRNAs often promote EMT by modulating chromatin structure or acting as miRNA sponges (24, 87, 88). Therapeutic strategies aimed at restoring tumor-suppressive ncRNAs or inhibiting oncogenic ncRNAs are being actively investigated using tools such as antagomirs, antisense oligonucleotides, miRNA mimics, and CRISPR-Cas9-based approaches. However, safe and efficient delivery remains a major challenge, and lipid nanoparticles (LNPs) and ligand-targeted carriers are being researched and developed to address this limitation (85).

Another promising avenue lies in targeting post-translational regulators of EMT. Enzymes such as Hakin-1, FBXW7, and USP27X influence the stability and activity of EMT TFs through protein degradation pathways, offering additional points for therapeutic intervention. Moreover, modulating the TME represents another approach to limiting EMT activation, with strategies targeting fibroblast-derived signals and immune-cell-mediated cytokine loops (89–91). Because EMT can influence tumor–immune interactions, combining EMT-targeted strategies with immunotherapy may enhance treatment responsiveness (92).

Recent studies have underscored the role of metabolic reprogramming in the EMT. Cancer cells undergoing EMT exhibit altered glucose, lipid, and amino acid metabolisms, supporting their survival and migration in hostile microenvironments (93). Metabolic inhibitors, including 2-deoxyglucose (2-DG), L-tetrahydro-2-furoic acid (L-THFA), and 4-methylumbelliferone, have demonstrated the potential to block EMT-associated metabolic shifts (94, 95). Repurposing such agents, many of which have been evaluated in other clinical contexts, could provide a feasible route for targeting the EMT with reduced toxicity. Epalrestat, which is currently undergoing Phase II trials for triple-negative breast cancer, exemplifies this strategy (96). These metabolic inhibitors are also being investigated as adjuncts to standard chemotherapy or immunotherapy to overcome the resistance associated with EMT.

EMT exists along a phenotypic continuum, and emerging evidence indicates that intermediate or hybrid EMT states, characterized by the co-expression of epithelial and mesenchymal markers, possess heightened metastatic and adaptive potential. Consequently, indiscriminate inhibition of EMT may inadvertently promote MET, enabling disseminated cells to reacquire epithelial traits conducive to secondary tumor formation (41).

EMT is closely associated with cancer stem cell (CSC) traits. EMT can endow cells with stem-like properties, including self-renewal and resistance to apoptosis, which contribute to tumor initiation, relapse, and poor clinical outcomes (97). Thus, targeting EMT may reduce the stemness of residual tumor cells and limit recurrence. Several clinical studies have begun to explore EMT-modulating therapies, including the TGF-β receptor I inhibitor, galunisertib, which has undergone early phase trials in combination with chemotherapeutics (98). Although the data remain preliminary, these efforts represent the initial steps in translating EMT research into clinical practice.

In addition to therapeutic interventions, targeting the EMT has been proposed as a chemopreventive strategy for high-risk individuals, particularly when the EMT contributes to early tumorigenic processes. However, caution must be exercised in this regard. The lack of robust clinical biomarkers for detecting and monitoring EMT progression, particularly in partial EMT states, limits translational success (93). Future research should focus on integrating liquid biopsy tools and single-cell technologies to monitor EMT dynamics in real time. CTCs, exosomal RNA, and spatial transcriptomics may help stratify patients and guide EMT-targeted therapies with greater precision. As our understanding of EMT biology continues to evolve, personalized multipronged approaches will be essential for overcoming the complexity of EMT-driven cancer progression and metastasis.

### 3.2 ECM inhibitors and anti-MMP therapies

Given its central role in tumor invasion and metastatic progression, the ECM has emerged as a therapeutic target. Among the enzymes that modulate ECM remodeling, MMPs, a family of zinc-dependent endopeptidases frequently overexpressed in tumors, are of particular interest (99, 100). Among the MMP family, isoforms such as MMP-2, MMP-3, MMP-9, and MMP-13 are particularly associated with invasion, angiogenesis, and metastatic niche formation, making them key candidates for selective therapeutic targeting (101).

Despite the well-established role of MMPs in cancer metastasis, therapeutic efforts to target them have faced significant challenges. Early generation broad-spectrum MMP inhibitors, such as batimastat and marimastat, were evaluated in clinical trials but were ultimately discontinued owing to limited efficacy and unacceptable musculoskeletal toxicity (102). These failures were not solely due to toxicity but also reflected an incomplete understanding of MMP biology at the time. While certain MMPs promote tumor invasion and angiogenesis, others maintain essential physiological functions in wound healing, immune modulation, and vascular homeostasis. Broad-spectrum inhibition disrupted this balance, leading to off-target effects and a narrow therapeutic window. Moreover, most clinical trials administered MMP inhibitors in late-stage cancers, where tumors were already well-established and vascularized (98). However, current evidence suggests that MMPs exert their most critical functions during the early phases of tumor progression, including local invasion and stromal remodeling. This treatment stage mismatch likely contributed to the lack of therapeutic benefit observed in advanced disease settings. Additionally, the absence of predictive biomarkers and insufficient stratification of patients whose tumors were truly MMP-driven may have further obscured potential responses (103). These drawbacks are largely attributed to the lack of specificity for individual MMP isoforms and the essential physiological roles played by MMPs in normal tissue remodeling and wound healing. Consequently, more recent strategies have focused on improving selectivity and minimizing systemic side effects. For instance, targeting specific MMPs associated with distinct stages of metastasis, such as MMP-2 in tumor invasion or MMP-9 in angiogenesis, offers a more refined approach. In a preclinical model, combining hemoglobin-loaded liposomes with chemotherapeutic agents effectively reduced MMP-2 expression and attenuated tumor invasiveness, suggesting that the indirect modulation of MMP activity through combination strategies may have therapeutic potential (104).

Beyond MMPs, LOX family enzymes have emerged as additional therapeutic targets due to their role in ECM stiffening and metastatic dissemination previously described in this review. Inhibitors of LOX are currently under investigation for their ability to prevent PMN formation and reduce metastatic burden (105).

Given the diverse and context-dependent functions of ECM remodeling in metastasis, therapeutic inhibition must be carefully optimized to balance efficacy with systemic safety. Because ECM remodeling occurs early and continuously during tumor progression, the timing of intervention is likely critical for clinical success. Emerging technologies such as ECM-targeted imaging and proteomic profiling may improve patient stratification and accelerate the translation of ECM-modulating therapies (106).

Ultimately, although direct inhibition of the ECM remains a challenging therapeutic frontier, it offers considerable promise when integrated into comprehensive anti-metastatic strategies. Selective targeting of matrix-degrading enzymes in combination with systemic treatments or nanotechnology-based delivery systems represents a next-generation approach to disrupting the physical and biochemical mechanisms that facilitate tumor dissemination and colonization.

### 3.3 Targeting hypoxia pathways

Hypoxia within the TME is a hallmark of solid malignancies and is closely linked to metastatic progression and resistance to conventional therapies. This adaptive response is largely mediated by HIF-1α and HIF-2α, which regulate transcriptional programs that promote survival, invasion, angiogenesis, and immune evasion (107, 108).

Considering its profound impact on tumor biology and clinical outcomes, hypoxia has emerged as a rational and validated target for therapeutic intervention (109). One widely explored approach involves inhibition of HIF signaling. Pharmacological strategies for blocking HIF function target various steps, such as protein stability, dimerization, transcriptional activity, and downstream gene expression (110). Agents including PX-478, digoxin, acriflavine, echinomycin, and bortezomib have shown preclinical activity in suppressing HIF-1α signaling, with some advancing to early-phase clinical trials (111–113). Specific inhibitors of HIF-2α, such as PT2385 and PT2977, have also demonstrated efficacy in hypoxia-driven tumors like renal cell carcinoma (114). Moreover, repurposed compounds, such as high-dose vitamin C and quercetin, interfere with HIF-mediated transcription, offering alternative strategies for modulating hypoxic responses (115, 116).

In addition to directly targeting HIFs, the development of hypoxia-activated prodrugs (HAPs) has provided a selective means of eradicating hypoxic tumor cells. These bioreductive agents remain inactive under normoxic conditions but are metabolically activated in hypoxic regions, enabling preferential toxicity to oxygen-deprived cancer cells (116). Examples include tirapazamine, PR-104, AQ4N, TH-302 (evofosfamide), and SN30000. Despite encouraging results in early phase trials, some HAPs have failed in phase III studies, partly owing to challenges in identifying suitable patient populations and achieving sufficient drug penetration in deeply hypoxic tumor zones. Nevertheless, HAPs continue to hold promise, particularly in targeting micrometastases and dormant cancer cells located in severely hypoxic niches (117).

Another avenue involves increasing oxygen availability in tumors through systemic reoxygenation strategies or localized oxygen delivery systems (116, 117). For instance, hyperbaric oxygen therapy has been shown to transiently improve tumor oxygenation and enhance the efficacy of radiation and immune checkpoint inhibitors (ICIs) (118). More advanced approaches include the use of oxygen-generating nanoparticles and bioengineered carriers such as catalase-loaded vesicles, which release oxygen directly into the hypoxic microenvironment (116). These strategies alleviate hypoxia and modulate the tumor vasculature and immune landscape, increasing the infiltration of cytotoxic T cells and reducing regulatory T cells and myeloid-derived suppressor cells (119).

In addition to HIF signaling and oxygen delivery, hypoxia drives tumor progression through metabolic reprogramming, ECM remodeling, and immune modulation. Hypoxia-induced metabolic shifts, such as increased glycolysis, glutamine metabolism, and lipid biosynthesis, support cancer cell survival in hostile environments and facilitate distant organ colonization (120, 121). Targeting these pathways, for instance, by inhibiting PDK1 or creatine kinase activity, may limit the metastatic capacity of hypoxia-adapted cells (120). Hypoxia also promotes ECM degradation and alignment through the upregulation of MMPs and LOX family enzymes (e.g., LOX and LOXL2), which facilitate cancer cell invasion and the establishment of premetastatic niches. In this context, digoxin- and LOX-directed antibodies have shown potential in preclinical models by limiting metastatic spread (122).

Crucially, hypoxia contributes to immune evasion by upregulating immune checkpoints such as PD-L1 via HIF-1α and fostering an immunosuppressive TME. Targeting these effects through combined oxygenation, HIF inhibition, and the blockade of downstream pathways, such as the adenosine A2A receptor axis, has been proposed to improve immunotherapy outcomes (123). Engineering immune cells, such as NK cells, to better withstand hypoxic stress is also under investigation as a means of overcoming hypoxia-induced resistance (124, 125).

Nanotechnology has further enhanced the ability to precisely target hypoxia. Nanoparticles can be engineered to deliver antihypoxic agents, siRNAs, or HAPs specifically to oxygen-deficient tumor regions, thereby improving the therapeutic index while minimizing off-target toxicity (116, 126). Red blood cell membrane-coated nanoparticles have been used to co-deliver chemotherapy and oxygen, while manganese dioxide nanoparticles have demonstrated dual action by alleviating hypoxia and suppressing HIF-1α expression (126, 127).

Despite these promising strategies, several major challenges remain. One key limitation of clinical translation is the heterogeneity of hypoxia across tumors and patients. Standardized, noninvasive methods to measure and stratify tumor hypoxia are lacking, rendering patient selection difficult in clinical trials. Advanced imaging techniques, such as positron emission tomography (PET) with hypoxia-sensitive tracers, are under development and may improve patient stratification (128). Additionally, the complexity of the hypoxia-driven transcriptional landscape, redundancy in survival pathways, and potential toxicity to normal tissues under physiological hypoxia must be addressed through refined therapeutic designs and combination regimens (116).

### 3.4 Inhibiting angiogenesis

Angiogenesis, the generation of new vasculature from pre-existing vessels, is a critical driver of tumor growth and metastatic dissemination. Tumors exploit angiogenic signaling to secure oxygen and nutrient supply, eliminate metabolic waste, and establish routes for invasion (129–131). Among the mediators of tumor angiogenesis, vascular endothelial growth factor A (VEGF-A) and its receptor VEGFR-2 constitute the principal signaling axis that regulates endothelial proliferation, migration, and survival. Elevated VEGF activity correlates with increased vascular density, aggressive tumor behavior, and poor prognosis, establishing the VEGF/VEGFR pathway as a central target for anti-angiogenic cancer therapy (131, 132).

#### 3.4.1 Anti-VEGF therapies

Therapeutic strategies targeting angiogenesis fall into three principal categories: ligand-neutralizing agents, VEGF receptor-directed agents, and receptor tyrosine kinase inhibitors (TKIs) (131). Ligand-neutralizing antibodies such as bevacizumab, the first Food and Drug Administration (FDA)-approved anti-angiogenic drug, bind VEGF-A and prevent its interaction with VEGFR-1 and VEGFR-2, thereby suppressing neovascularization and reducing vascular permeability, two key drivers of metastatic spread. Bevacizumab has demonstrated clinical benefit in metastatic colorectal cancer, non-small-cell lung cancer, glioblastoma, and other malignancies (133).

VEGF receptor-targeting agents, including the fusion protein aflibercept (VEGF-trap), act as decoy receptors that sequester VEGF-A, VEGF-B, and placental growth factor (PlGF), producing broader blockade of pro-angiogenic signaling (134). In contrast, TKIs such as sunitinib, sorafenib, and pazopanib inhibit the intracellular kinase activity of VEGFRs and related angiogenic receptors, thereby curtailing endothelial proliferation and metastatic vascular support (129–132, 135).

Short-term anti-VEGF therapy can transiently normalize aberrant tumor vasculature, reducing leakiness, improving perfusion, and enhancing drug and oxygen delivery. This “vascular normalization” window enhances the efficacy of chemotherapy, radiotherapy, and immunotherapy, but is transient and dose-dependent, requiring precise scheduling to exploit optimally (136).

Nonetheless, durable clinical responses remain limited by adaptive resistance mechanisms, including compensatory up-regulation of alternative angiogenic pathways and recruitment of pro-angiogenic myeloid cells. These limitations underscore the rationale for integrating VEGF-targeted agents with cytotoxic, immunomodulatory, or other anti-metastatic therapies to achieve sustained therapeutic benefit.

#### 3.4.2 Limitations and resistance mechanisms of anti-VEGF

Despite the initial clinical benefit, anti-VEGF therapies frequently encounter intrinsic or acquired resistance, leading to transient responses and poor long-term survival (137, 138). Resistance arises through several mechanisms. Tumors circumvent VEGF blockade by activating parallel pro-angiogenic signaling networks involving PDGF, bFGF, Ang-1/2, PlGF, and VEGF-D, the latter stimulating VEGFR-3-mediated lymphangiogenesis even under VEGF-A inhibition (134, 137, 139, 140). In addition, tumors can adopt alternative vascularization strategies such as vessel co-option, vasculogenic mimicry, and intussusceptive angiogenesis, all of which sustain perfusion independent of classical VEGF signaling (134, 138, 141–143).

Anti-angiogenic therapy also remodels the TME, often aggravating hypoxia and stabilizing HIF-1α, which, in turn, re-induces angiogenic and pro-survival genes. Hypoxia recruits immunosuppressive myeloid and regulatory cells (MDSCs, TAMs, and Tregs) and drives desmoplastic ECM deposition that limits drug penetration, collectively reinforcing therapeutic resistance (144, 145). Additional contributors include endothelial heterogeneity, MDR protein overexpression, lysosomal degradation of TKIs, and angiogenic gene polymorphisms affecting drug response (131, 136, 145).

The clinical utility of VEGF blockade is further constrained by toxicity, as VEGF is integral to normal vascular maintenance. Adverse events such as hypertension, proteinuria, hemorrhage, and impaired wound healing frequently necessitate dose reduction or discontinuation, narrowing the therapeutic window (146, 147). Moreover, rebound hypoxia following vessel regression can paradoxically promote EMT and metastatic dissemination, illustrating the dualistic nature of angiogenesis suppression (146).

To address these limitations, combination regimens are increasingly employed. Integrating VEGF inhibitors with chemotherapy enhances drug delivery and cytotoxicity, while combinations with immune checkpoint blockade reprogram the immunosuppressive tumor milieu and convert “cold” tumors to “hot” ones more amenable to immunotherapy (144, 148–150). Dual angiogenic blockade (e.g., VEGF plus Ang-2 or PDGFR inhibition) and rational scheduling to exploit transient vascular normalization further improve outcomes (151–154). Collectively, these advances underscore that durable benefit requires targeting angiogenesis within multimodal frameworks guided by predictive biomarkers and precision-medicine principles.

### 3.5 Immunotherapies for metastatic cancer

Immunotherapy has revolutionized metastatic cancer treatment by enabling the body’s immune system to detect and destroy tumor cells. Unlike conventional therapies that directly target tumor proliferation or vascularization, immunotherapy modulates the immune response to achieve durable control or eradication of malignancies. Among the most significant advances are ICIs and chimeric antigen receptor (CAR) T-cell therapies, both of which have demonstrated promise for the treatment of advanced cancers, although each faces distinct challenges in the metastatic setting.

#### 3.5.1 Immune checkpoint inhibitors

Immune checkpoints function as vital mechanisms that maintain self-tolerance while controlling the intensity of physiological immune reactions. However, tumor cells often utilize these pathways, particularly the CTLA-4 and PD-1/PD-L1 axes, to escape immune surveillance. ICIs function by disrupting these inhibitory signals, removing T-cell activation restrictions, and restoring antitumor immunity (155, 156).

The first checkpoint inhibitor to receive FDA approval was ipilimumab, an anti-CTLA-4 antibody that demonstrated survival benefits in metastatic melanoma. CTLA-4, expressed on Tregs and activated T cells, impairs immune priming by inhibiting the co-stimulatory signaling between CD28 and its ligands (CD80/CD86) in antigen-presenting cells. Ipilimumab counteracts immunosuppression by enhancing cytotoxic T lymphocyte (CTL) activity (157).

Subsequent therapeutic developments focused on the PD-1/PD-L1 axis. PD-1 is an inhibitory receptor expressed on activated T cells, whereas PD-L1 is overexpressed in tumors and stromal cells. These interactions suppress T-cell proliferation, cytokine production, and survival. PD-1 inhibitors such as nivolumab and pembrolizumab, and PD-L1 inhibitors including atezolizumab, durvalumab, and avelumab, have been approved for a broad spectrum of solid tumors, including metastatic melanoma, NSCLC, renal cell carcinoma, hepatocellular carcinoma, and urothelial carcinoma (158–160).

Nivolumab and ipilimumab combination therapy demonstrated superior results to monotherapy in metastatic melanoma patients through improved survival rates and sustained treatment outcomes (161, 162). Pembrolizumab, alone or in combination with chemotherapy, is the first-line treatment option for metastatic NSCLC. ICIs have also shown benefits in colorectal cancers with high microsatellite instability (MSI-H) or mismatch repair deficiency (dMMR), which are characterized by high tumor mutational burden and neoantigen load, factors that enhance immunogenicity (162).

Despite their potential for transformation, ICIs are not universally effective. A substantial proportion of patients exhibit primary resistance, and many experience relapse. Resistance mechanisms include low tumor immunogenicity; absence or exclusion of T cells from the TME; and compensatory immunosuppressive mechanisms involving MDSCs, Tregs, or alternative checkpoint pathways. Moreover, immune-related adverse events (irAEs), ranging from dermatitis and colitis to endocrinopathies and pneumonitis, can necessitate treatment discontinuation and immunosuppressive interventions (158, 163).

#### 3.5.2 CAR-T cell therapies and their challenges in metastasis

The development of CAR T-cell therapy has led to significant progress in immuno-oncology, particularly in the treatment of hematological conditions. This approach involves the *ex vivo* genetic modification of autologous T cells to express CARs that target specific tumor-associated antigens. Upon reinfusion, the engineered cells recognize and destroy malignant cells in a major histocompatibility complex (MHC)-independent manner. The application of CD19-targeted CAR-T cells has proven highly successful in treating B-cell malignancies, achieving complete remission in many patients with acute lymphoblastic leukemia (ALL) and diffuse large B-cell lymphoma (DLBCL) (164).

However, translating the success of CAR-T therapy to solid tumors, especially in metastatic settings, has proven more difficult. As previously discussed, the immunosuppressive and structurally complex TME remains a major obstacle to effective CAR-T therapy. Factors such as cytokine-mediated suppression, cellular inhibitors, and hypoxia collectively impair T-cell activation, infiltration, and persistence within solid tumors. Additionally, the abnormal vasculature and dense ECM of solid tumors limit the effective trafficking, infiltration, and survival of CAR-T cells at tumor sites (165).

Antigen heterogeneity and immune escape are also obstacles. Unlike hematologic cancers, in which a uniform and lineage-restricted antigen such as CD19 can be targeted, solid tumors often exhibit variable and heterogeneous antigen expression. This variability can result in antigen-loss variants or selection of resistant tumor clones, leading to treatment failure. Furthermore, many tumor-associated antigens are expressed at low levels in normal tissues, increasing the risk of on-target off-tumor toxicity, a potentially life-threatening complication (166).

Therefore, various methods have been developed to overcome these limitations. Strategies include engineering CAR-T cells with dual or tandem CARs to target multiple antigens, using armored CAR-T cells that secrete immune-stimulating cytokines, and combining CAR-T therapy with ICIs or antiangiogenic agents to improve T-cell infiltration and function. Locoregional delivery approaches such as intratumoral or intra-arterial injections have also been explored to enhance tumor-specific accumulation and reduce systemic toxicity (165). Additionally, novel delivery platforms, such as biomaterial scaffolds and CAR-macrophage (CAR-M) therapies, are under investigation for their ability to improve tumor penetration and modulate the suppressive TME (167).

Despite these advancements, CAR T-cell therapy for metastatic solid tumors remains in its early clinical stages. The need for improved antigen specificity, enhanced cell persistence, and resistance to immunosuppression continues to drive innovation in CAR engineering, delivery strategies, and combination regimens. As our understanding of tumor–immune system interactions deepens, the integration of CAR-T cell therapy into metastatic cancer treatment models holds considerable promise, particularly when combined with other immunomodulatory and cytotoxic modalities (168).

### 3.6 Recent advancements in drug delivery systems

Effective management of metastatic disease requires drug delivery systems that directly target the biological and physical pathways enabling invasion and colonization. Conventional formulations often fail because metastatic niches are protected by desmoplastic extracellular matrices, aberrant and poorly perfused vasculature, and immunosuppressive microenvironments that restrict penetration and foster cellular dormancy (169–171). Additional challenges arise from organ-specific barriers such as the blood–brain barrier, from physical forces including elevated interstitial pressure and solid stress that impair convective transport, and from pharmacokinetic limitations such as rapid clearance and multidrug resistance. As detailed in earlier sections, features such as ECM remodeling, vascular disorganization, and immune suppression actively contribute to metastatic progression rather than serving as passive barriers; therefore, delivery platforms must be designed to counteract these mechanisms.

Recent nanotechnology-based systems integrate molecular and physical design principles to overcome these constraints. Immune-modulating nanoparticles reprogram suppressive immune and stromal cells within the metastatic niche; ECM-degrading or shape-adaptive carriers penetrate dense stroma; and stimuli-responsive formulations release drugs in response to pH, enzymatic activity, or hypoxia—conditions characteristic of invasive lesions (172, 173). Ligand conjugation and biomimetic surface coatings improve organ-specific targeting, prolong systemic circulation, and enhance therapeutic retention. Collectively, these advances couple drug-delivery engineering with the molecular hallmarks of metastasis, providing a framework for selective and sustained eradication of DTCs.

#### 3.6.1 Precision medicine approaches, including nanotechnology, lipid carriers, and targeted delivery systems

The most clinically validated nanocarriers include lipid-based systems such as liposomes, solid lipid nanoparticles (SLNs), and nanostructured lipid carriers (NLCs). Liposomes consist of phospholipid bilayers surrounding an aqueous core and can encapsulate both hydrophilic and hydrophobic drugs (174). Modifications such as PEGylation improve the circulation half-life by evading opsonization and phagocytosis, while ligand conjugation ensures active targeting (173). The success of liposomal doxorubicin (Doxil^®^), the first FDA-approved nanodrug, underscores the translational potential of this platform. In addition, LNPs have become prominent vehicles for nucleic acid delivery, particularly mRNA delivery, offering applications in both cancer therapeutics and immunization strategies. These carriers demonstrate high transfection efficiency, endosomal escape, and safety profiles, making them ideal for delivering gene regulatory sequences to metastatic cells (175).

Targeted drug delivery is the cornerstone of nanoparticle drug delivery system (NDDS) functionality and is achieved through both passive and active mechanisms. Passive targeting uses the enhanced permeability and retention (EPR) effect, a phenomenon characterized by leaky tumor vasculature and impaired lymphatic drainage, permitting the preferential accumulation of nanoparticles at tumor sites. Active targeting involves the surface functionalization of nanoparticles with ligands such as antibodies, peptides, aptamers, or vitamins that bind selectively to receptors overexpressed on tumor cells, thereby enhancing cellular uptake and retention. Such precision is vital in the metastatic context, where DTCs often reside in complex or inaccessible places, such as the brain, bone marrow, or lymphatic system. Active targeting facilitates greater intracellular uptake and enhances therapeutic efficacy by maintaining localized drug concentrations, thereby reducing the need for high systemic doses that contribute to toxicity (176–178).

Another approach for achieving precise drug delivery is the development of stimuli-responsive nanocarriers. These advanced systems are designed to release therapeutic payloads in response to specific endogenous stimuli, such as acidic pH, high levels of glutathione, tumor-associated enzymes, or exogenous triggers including temperature, magnetic fields, ultrasound, or light. This responsiveness enables spatiotemporal control of drug release, providing on-demand activation at the tumor site while minimizing off-target effects. Such systems are particularly valuable for treating metastases located in organs with sensitive microenvironments, such as the brain or lungs (179). By enhancing penetration, selective accumulation, and controlled release within metastatic niches, these precision delivery systems directly address the microenvironmental barriers that sustain invasion, dormancy, and colonization.

#### 3.6.2 Specific examples such as SNA and amphiphile-based systems

Spherical nucleic acids (SNAs) represent a next-generation class of nanotherapeutics constructed by densely arranging DNA, RNA, or miRNA strands around a nanoparticle core, such as gold, polymers, or liposomes. This three-dimensional architecture enhances cellular uptake and nuclease resistance. Their structural design facilitates the efficient delivery of gene regulatory agents (e.g., siRNA or miRNA) to silence oncogene expression, with advanced formulations demonstrating high-density duplex loading and improved endosomal escape for sustained knockdown effects (180).

In addition to gene silencing, SNAs exhibit a strong immunotherapeutic potential. Immunostimulatory SNAs functionalized with Toll-like receptor (TLR) agonists, such as CpG oligonucleotides, activate dendritic cells and induce robust T cell-mediated antitumor immunity. Hybridized SNAs that co-deliver tumor antigens and immune adjuvants have shown enhanced T-cell activation and tumor regression in preclinical models. Liposomal SNAs, which are constructed by combining amphiphilic liposomal cores with surface-bound nucleic acids, exemplify the convergence of gene modulation and immune activation strategies. These systems have demonstrated the ability to cross the blood–brain barrier, making them promising candidates for targeting brain metastases. Early-phase clinical studies have confirmed their safety, and ongoing efforts employing artificial intelligence and high-throughput screening aim to further refine their therapeutic efficacy through optimized structure–function relationships (180, 181).

Polymeric micelles and amphiphile-based nanocarriers have also gained attention in metastatic cancer therapy. These structures are formed by the self-assembly of amphiphilic block copolymers, creating core–shell architectures capable of encapsulating hydrophobic drugs. Their small size enhances tumor penetration and lymphatic transport. A well-known example is Genexol-PM^®^, a paclitaxel-loaded polymeric micelle formulation that has been approved for clinical use in several countries, including South Korea and parts of Asia, although it has not yet received FDA approval in the United States (182). Further refinement has led to the development of peptide amphiphile micelles (PAMs), which incorporate targeting ligands or cell-penetrating peptides into the micellar structure to achieve receptor-specific delivery and enhance cellular uptake (183). Collectively, these nucleic acid and amphiphile-based nanocarriers address key challenges in metastatic cancer by enabling gene regulation, immune activation, and efficient drug transport across physiological barriers such as the blood–brain barrier, thereby improving therapeutic access to disseminated and treatment-resistant tumor cells.

#### 3.6.3 Clinical translation and ongoing challenges

The integration of nanotechnology with precision medicine is transforming metastatic cancer treatment by enabling targeted and personalized drug delivery. Clinical examples include PEGylated liposomal doxorubicin with trastuzumab for HER2-positive breast cancer (184). Bone-targeting alendronate nanoparticles (185) and brain-targeting intranasal nanocarriers (186) are under active preclinical and translational investigation. These strategies demonstrate the adaptability of nanoscale delivery systems to overcome site-specific challenges in metastatic treatments. By addressing the biological and physiological barriers that characterize metastatic niches, such as vascular heterogeneity, immune suppression, and organ specific permeability, these targeted systems exemplify how precision nanomedicine can directly influence invasion, colonization, and treatment resistance.

Despite promising outcomes, translational challenges remain significant. Drawbacks such as inconsistent nanoparticle accumulation, variable drug ratios, scale-up difficulties, and unintended deposition in non-target organs, such as the liver and spleen, continue to impede widespread clinical application. Addressing these challenges requires refined nanoparticle design, improved manufacturing protocols, and more precise patient stratification (187).

**Figure 6:**
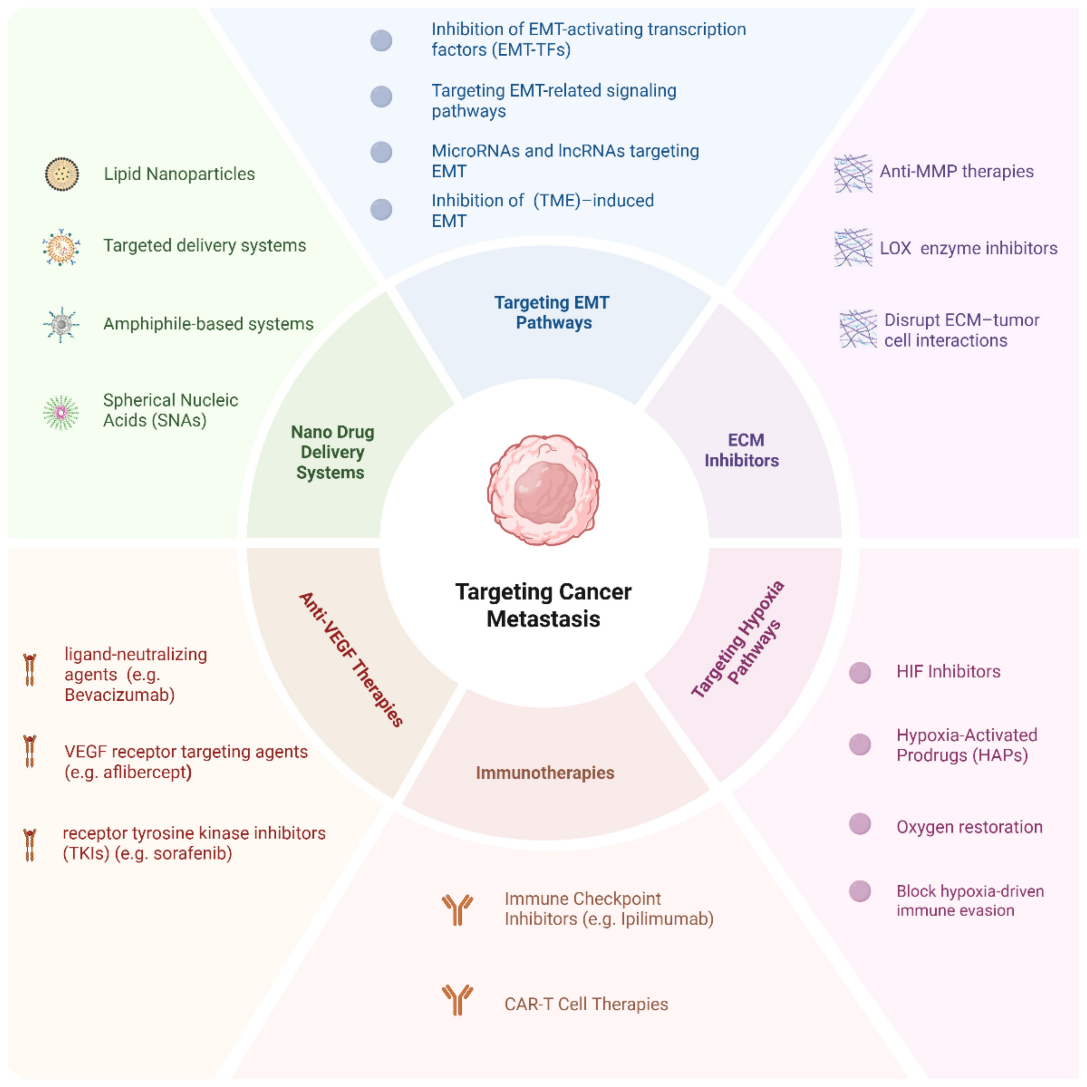
Targeting cancer metastasis: a comprehensive overview of therapeutic strategies. This diagram illustrates the six principal therapeutic categories aimed at combating cancer metastasis.

Recent innovations, including SNAs, peptide-functionalized amphiphilic systems, and stimuli-responsive carriers, demonstrate the evolving sophistication of nanomedicines. These platforms enhance drug solubility, stability, and release kinetics, while minimizing systemic toxicity. With interdisciplinary research bridging the fields of materials science, oncology, and bioengineering, the development of intelligent patient-specific nanomedicines is reshaping metastatic cancer therapy. With continued advancements, these next-generation systems hold great promise for delivering safer, more effective, and highly individualized treatments. **Figure 6** provides a comprehensive overview of the key interventions discussed in this section.

## 4 Emerging technologies and innovations

### 4.1 Multi-omics approaches

EMT transition, cell adhesion, angiogenesis, and lymphangiogenesis are among the various multistep processes involved in cancer cell invasion and metastasis (169). Tumor heterogeneity, which encompasses genetic, epigenetic, and metabolic elements that drive cancer progression, complicates the identification and prevention of recurrence (188). Although conventional single-omics approaches are valuable, they provide limited perspectives and are inadequate for capturing the complexity of cancer (189, 190). This limitation is particularly critical in metastasis, where coordinated alterations across genomic, transcriptomic, and proteomic networks drive invasion, immune evasion, and colonization.

These limitations highlight the need for more thorough methods to provide better knowledge of cancer biology (190). By capturing the complex interactions between molecular processes, multi-omics techniques, including genomics, transcriptomics, proteomics, and metabolomics, offer a more thorough understanding of cancer biology (190).

Through this systems-level integration, researchers can trace how molecular heterogeneity translates into metastatic phenotypes and treatment resistance. Integrating multi-omics data offers the potential to identify unique biomarkers for early detection, prognosis, and therapeutic responses. Additionally, multi-omics facilitates the identification of new therapeutic targets and strategies to combat cancer invasion and metastasis (191, 192). Nonetheless, the analysis of multi-omics data to identify trends and links between several data types depends on advanced computational techniques and machine learning algorithms, which provide insights into the fundamental causes of cancer progression (188, 193).

Additionally, proteomic profiling highlights active signaling pathways and post-translational modifications related to cell migration, invasion, and ECM remodeling, further enhancing our understanding of these processes (194).

**Figure 7:**
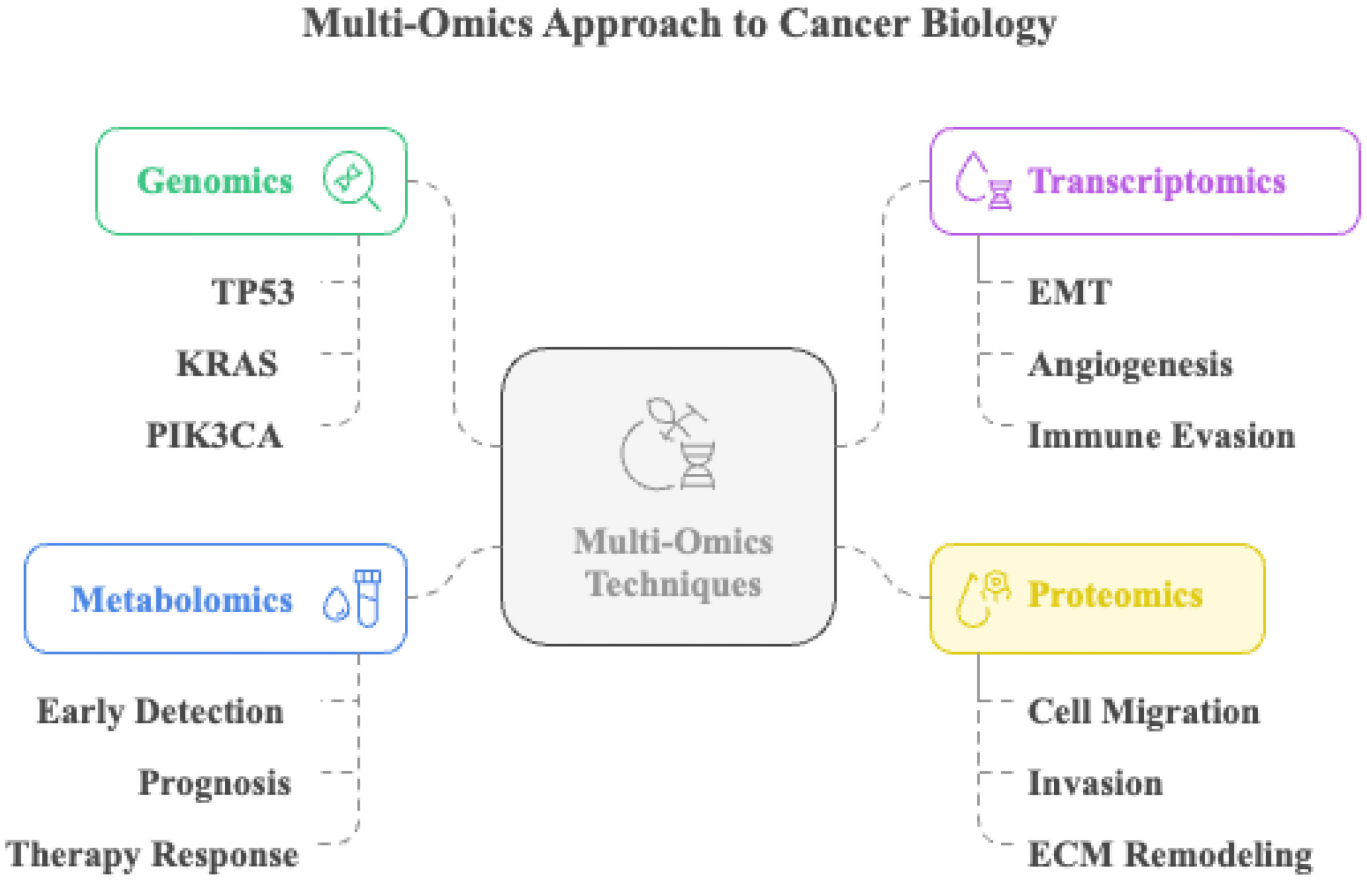
Multi-omics approach to cancer biology. This illustration depicts the integration of multi-omics techniques, including genomics, transcriptomics, metabolomics, and proteomics, to comprehensively investigate cancer biology. Genomic profiling identifies frequently mutated genes, such as TP53, KRAS, and PIK3CA. Transcriptomic analysis reveals gene expression changes associated with epithelial–mesenchymal transition (EMT), angiogenesis, and immune evasion. Metabolomic profiling aids in early detection, prognosis, and evaluation of therapy response. Proteomic data provide insights into mechanisms like cell migration, invasion, and extracellular matrix (ECM) remodeling.

Integrative multi-omics platforms have improved our knowledge of metastatic processes and offered new targets for pathway-specific therapies (195). Genomic sequencing has revealed mutations in oncogenes and tumor suppressors such as TP53, KRAS, and PIK3CA, which are associated with metastatic potential in many malignancies (196). With the growing importance of lncRNAs and microRNAs, transcriptomic studies have shown that they alter the expression of genes that control the EMT, angiogenesis, and immune evasion (197). Beyond enriching our mechanistic understanding, multi-omics approaches are now demonstrating direct clinical utility in monitoring metastatic evolution, detecting minimal residual disease (MRD), and guiding personalized therapeutic strategies. For instance, longitudinal tumor-informed ctDNA profiling coupled with genomic and transcriptomic modeling, as shown in the TRACERx lung cancer program, enables real-time tracking of subclonal expansion and metastatic seeding, identifying relapse months before radiological detection (198, 199). Likewise, plasma-only multi-omics MRD assays that integrate somatic variants with epigenomic (methylation) signatures have increased sensitivity for detecting micrometastasis and residual disease in early-stage breast and colorectal cancer, supporting risk-adapted adjuvant therapy and intensified surveillance (200, 201). In terms of patient stratification, proteogenomic analyses (e.g., CPTAC) have revealed pathway activation states and phospho-signaling dependencies not predictable from genomics alone, informing therapeutic prioritization and resistance mechanisms (202, 203). Complementing this, the WINTHER clinical trial demonstrated that incorporating transcriptomic profiling alongside tumor genomics increased the proportion of patients matched to targeted therapy and improved clinical benefit, underscoring the translational relevance of integrative multi-omics in precision oncology decision-making (204). Emerging single-cell and spatial multi-omics further allow resolution of rare disseminated clones and metastatic niche interactions, holding promise for earlier intervention and rational combination treatment design (205). The multi-omics techniques are summarized in **Figure 7**.

### 4.2 Examination of single cells

Single-cell analysis has become an invaluable tool for interrogating intratumoral heterogeneity and the TME to elucidate the dynamic mechanisms underlying cancer invasion and metastasis (206). Conventional bulk profiling averages signals across large cell populations, thereby masking rare but clinically consequential subclones that drive metastatic progression (206). In contrast, single-cell approaches enable the resolution of gene expression, protein activity, and epigenetic states at the individual-cell level, allowing the identification of discrete metastatic subpopulations and the molecular pathways that sustain their invasive phenotypes (207).

Emerging single-cell technologies, including spatial transcriptomics and scRNA-seq, now provide high-resolution maps of tumor ecological structure (208). These approaches have uncovered rare cell states, such as cancer stem-like subpopulations and hybrid E/M phenotypes, which disproportionately contribute to dissemination and therapeutic resistance (209). Additionally, single-cell analysis has delineated complex interactions between malignant cells and immune or stromal elements in the TME, clarifying how microenvironmental cues reinforce invasion and support metastatic colonization (210).

Beyond mechanistic insight, single-cell platforms are increasingly demonstrating direct clinical relevance. Single-cell DNA and multi-omics analyses can reconstruct patient-specific clonal evolution across primary and metastatic lesions and during therapy, enabling the identification of emergent drug-tolerant subclones for timely therapeutic intervention (206–208). Moreover, scRNA-seq profiling of circulating or compartmentalized tumor cells allows early detection of micrometastasis, surpassing the sensitivity of conventional cytology, and supports longitudinal molecular disease monitoring (209–212). Finally, integration of single-cell tumor and immune signatures into machine-learning frameworks is advancing personalized treatment stratification, including predicting response to ICIs and prioritizing rational multi-target drug combinations (150). Together, these advancements position single-cell technologies not only as discovery tools but also as emerging clinical decision-support systems.

### 4.3 Bioinformatics and computer modeling

Bioinformatics and computational modeling have become indispensable tools for decoding the complex molecular and biophysical mechanisms that drive cancer invasion and metastasis. Machine learning models trained on multi-omics and clinical datasets can stratify patients according to metastatic risk and therapeutic response, improving prognostic precision (213, 214). The reconstruction of signaling networks and virtual drug testing *in silico* enables data-driven hypothesis generation, prioritization of anti-metastatic targets, and rapid evaluation of therapeutic strategies (215). These computational approaches allow mechanistic exploration of invasion-associated signaling pathways, clonal adaptation, and microenvironmental feedback loops that are difficult to capture experimentally.

By integrating genomics, transcriptomics, and proteomics, computational models clarify how molecular alterations converge to promote metastatic progression (169). In particular, the combination of multi-omics data with mathematical and machine learning frameworks has transformed metastasis research, accelerating the development of targeted and individualized treatments (216–219). Computational modeling also provides a quantitative foundation for understanding the mechanical determinants of metastasis. Mechanical features of the TME, including stiffness and stress, influence cell migration, invasion, and dissemination (220, 221). Simulating these forces *in silico* reveals how cancer cells adapt to mechanical constraints and how therapeutic interventions can be designed to modulate these dynamics, potentially reducing metastatic spread.

Bioinformatics complements these efforts by enabling large-scale interpretation of genomic and transcriptomic data to identify key genes, pathways, and biomarkers involved in metastatic progression (222). Through comparative expression and mutation analyses, bioinformatics tools can uncover upregulated and downregulated genes that regulate EMT, immune evasion, and organ-specific colonization. Translational bioinformatics extends this framework to clinical practice by integrating clinical, genetic, and molecular data to identify biomarkers that predict outcomes and therapy responses (223, 224). The development of advanced computational pipelines for next-generation sequencing and real-time molecular monitoring allows detection of resistance mechanisms and adaptation of treatment strategies (225).

**Figure 8:**
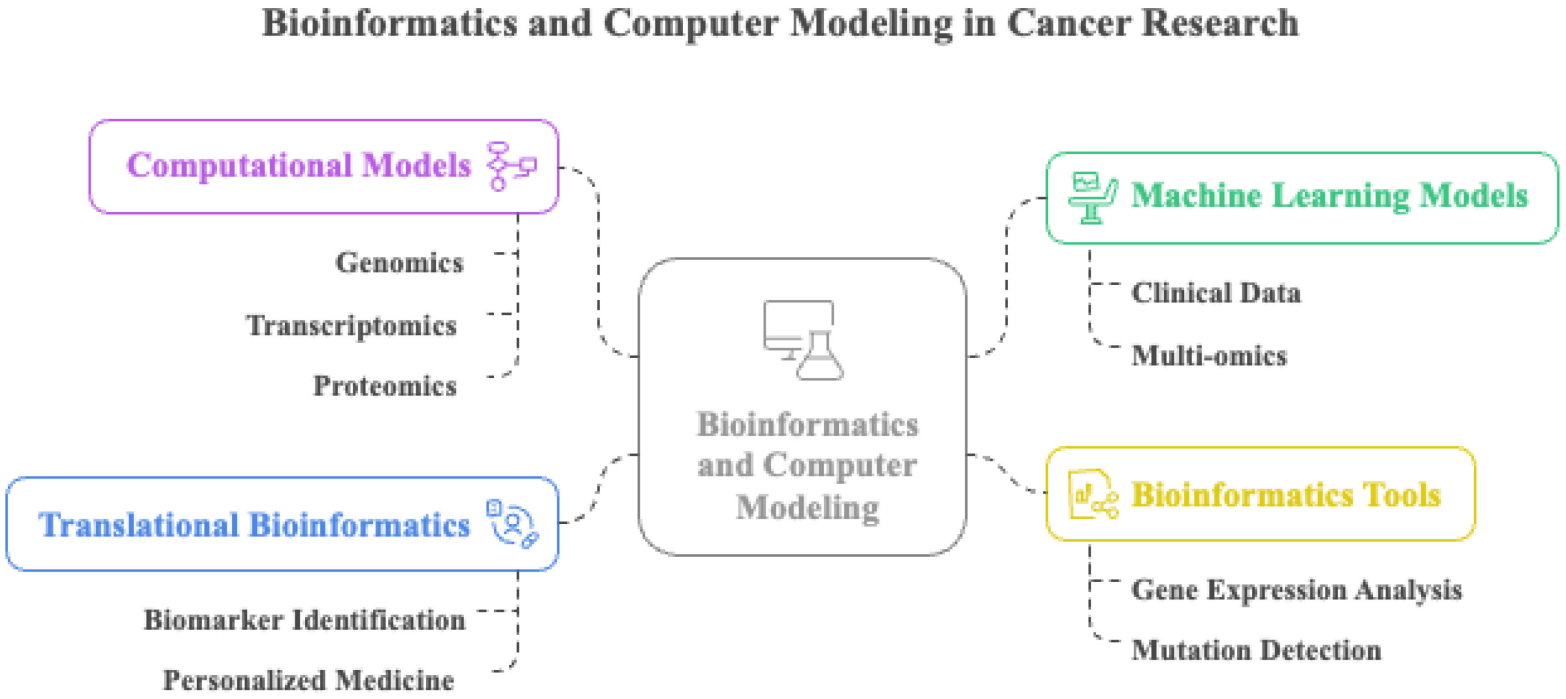
Integration of bioinformatics and computer modeling approaches in cancer research. The central framework combines four major domains: computational models, machine learning models, translational bioinformatics, and bioinformatics tools. These components work together to facilitate a system-level understanding of cancer biology and support precision oncology applications.

Together, the integration of bioinformatics and computational modeling bridges experimental biology with clinical oncology, revealing functional relationships between gene networks, mechanical properties, and metastatic phenotypes. This synergy not only enhances our mechanistic understanding of metastasis but also supports the development of precision therapies tailored to the molecular and physical characteristics of individual tumors. Key computational approaches are summarized in **Figure 8**.

### 4.4 Gene editing using CRISPR-Cas9

The advent of CRISPR-Cas9 technology has revolutionized cancer research by enabling precise and efficient genome editing. Derived from the adaptive immune system of bacteria and archaea, this tool allows targeted modification of DNA sequences within living cells, facilitating functional gene analysis and the development of novel therapeutic strategies (226, 227). In metastasis research, CRISPR-Cas9 provides a direct means to interrogate causal relationships between specific genes and metastatic behavior. Targeted editing of key regulators such as SNAI1, TWIST1, and MMP9 has clarified their roles in EMT, ECM remodeling, and invasion (226). By functionally dissecting these and related genes, CRISPR approaches reveal hierarchical networks that orchestrate invasion, dissemination, and colonization, thereby identifying potential therapeutic vulnerabilities that cannot be resolved through correlative omics analyses alone.

High-throughput CRISPR screens have further expanded this capability, uncovering novel regulators of cell invasion, survival in circulation, and secondary site colonization (227). Pooled knockout libraries allow systematic assessment of phenotypes such as drug resistance, dormancy, or immune evasion, providing functional validation of metastasis-associated pathways (228). Moreover, CRISPR-based tools are increasingly being integrated into combinatorial studies with transcriptomics and proteomics to map feedback loops that sustain metastatic persistence and therapeutic resistance.

Despite its transformative potential, several challenges limit the clinical translation of CRISPR-Cas9. Off target effects, immunogenicity, and inefficient delivery remain major obstacles that must be overcome to achieve safe and effective therapeutic application (229). Continued refinement of guide RNA design, delivery vectors, and editing precision will be essential for fully realizing CRISPR’s role in dissecting and therapeutically targeting the molecular circuitry of metastasis.

### 4.5 Liquid biopsy and biomarkers

The minimally invasive and unique approach of liquid biopsy shows promise for the dynamic surveillance of tumor metastasis. It could revolutionize cancer management by revealing the changing landscape of the disease. Unlike traditional tissue biopsies, which are fixed and localized, liquid biopsies can gather CTCs and cell-free DNA (cfDNA) from the blood. This helps track disease progression and the efficacy of treatments over time (230). In various metastatic tumors, this technique overcomes the drawbacks of traditional biopsies, which are invasive, expensive, and subject to sample bias (231). Liquid biopsies overcome these limitations by enabling real-time and comprehensive analysis of the cancer genome and tumor heterogeneity. In particular, circulating tumor DNA (ctDNA) analysis through liquid biopsy offers a viable alternative to traditional methods, enabling the detection of early recurrence and distinguishing tumor progression from pseudo-progression without the need for repeated surgical samples (232). The clinical value of liquid biopsy lies in its ability to identify actionable mutations in metastatic lesions that may not be present in the primary tumor (233). This enables a comprehensive and adaptable understanding of cancer pathology, supporting continuous biomarker monitoring, early detection of therapy resistance, and the discovery of new therapeutic targets.

Liquid biopsy not only enhances treatment monitoring but also accelerates drug development. By monitoring ctDNA levels during therapy, healthcare providers can promptly discover therapeutic responses or resistance and adjust the treatment plan (234). This approach is beneficial for targeted therapies, as it identifies resistance mutations early, allowing for therapeutic adjustments before clinical symptoms appear. Furthermore, liquid biopsies enable drug developers to identify predictive biomarkers and determine which patients are most likely to benefit from new treatments (235).

Although liquid biopsy has advanced, several challenges must be resolved before it can be widely employed in clinical settings. For instance, low levels of CTCs and cfDNA in the bloodstream, especially in the early stages of cancer, require sensitive and specific detection methods. Additionally, pre-analytical and analytical methods must be standardized, as sample collection, processing, and analysis can significantly impact outcomes. Furthermore, the incorporation of microfluidics in liquid biopsy platforms could enhance the sensitivity and efficiency of biomarker detection (236). Improved bioinformatics tools are also needed to differentiate tumor-specific signals from background noise in cfDNA sequencing. Additionally, to ensure responsible therapeutic application, ethical issues such as data privacy and unexpected findings must be carefully considered.

**Figure 9:**
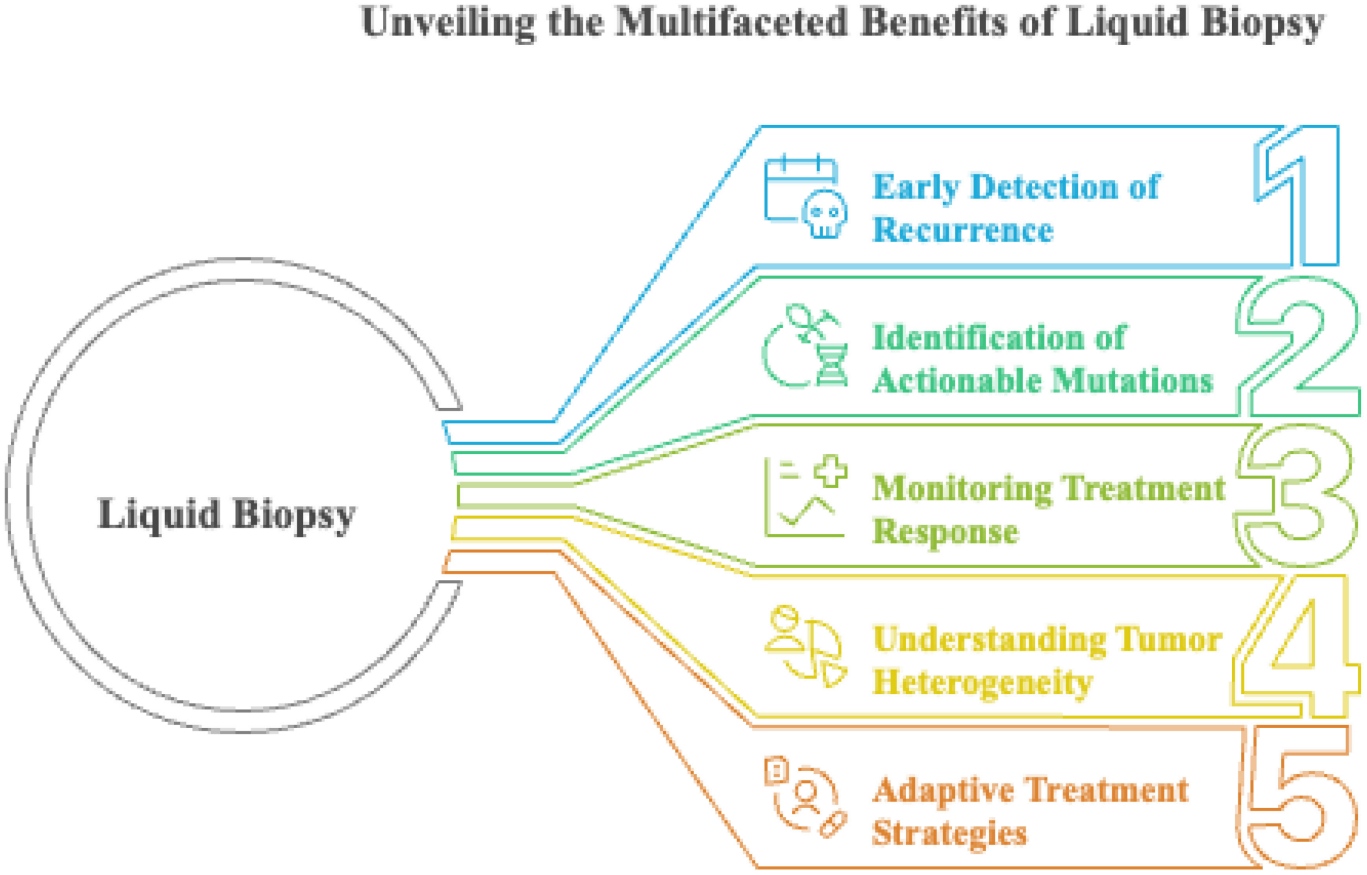
Unveiling the multifaceted benefits of liquid biopsy. This schematic illustrates five key clinical advantages of liquid biopsy in oncology.

New technologies and analytical methods are expected to further improve the sensitivity, specificity, and clinical relevance of liquid biopsies. Artificial intelligence and machine learning algorithms can identify patterns and correlations in complex multi-omics datasets that statistical methods might overlook (237). Including epigenetic indicators, such as DNA methylation and histone modifications, into liquid biopsy testing can help elucidate metastatic disease biology and uncover new treatment targets (238). Therefore, to make liquid biopsies more routine in clinical settings and improve outcomes for patients with metastatic cancer, future research should address these challenges and validate their clinical efficacy in large-scale, prospective trials (239). The multifaceted benefits of liquid biopsy are summarized in **Figure 9**.

## 5 Challenges, limitations, and future perspectives in understanding and treating cancer metastasis

As discussed earlier in this review, cancer metastasis accounts for most cancer-related deaths worldwide (240). Despite significant advances in cancer biology and therapeutics, metastasis remains a formidable challenge owing to its complex and multifactorial nature. The persistence of metastatic disease reflects the remarkable plasticity of cancer cells, their ability to co-opt host microenvironments, and the inadequacy of current models to capture the temporal and spatial dynamics of dissemination. This section explores the current gaps in our understanding of metastasis and its regulation, limitations of existing therapies, the promise of personalized medicine, the transformative potential of interdisciplinary collaboration, and the critical importance of patient diversity in clinical trials (241, 242).

### 5.1 Biological complexity and knowledge gaps

Despite major advances in characterizing the molecular pathways underlying invasion and metastasis, several fundamental uncertainties remain. Tumor heterogeneity within both primary and metastatic sites complicates the identification of consistent biomarkers and therapeutic targets (13). How clonal evolution, stromal remodeling, and immune modulation integrate over time to enable dissemination and colonization is still unclear (243).

Recent lineage-tracing and spatial-omics studies indicate that only specific tumor subclones possess the plasticity required for successful metastasis; however, the timing and selective pressures driving their emergence remain poorly resolved. The human relevance of PMN formation and its molecular mediators also remains insufficiently defined, as current understanding derives largely from experimental models lacking clinical validation (244–246).

Despite progress in targeting EMT-associated non-coding RNAs, their extensive cross-regulation and tissue-specific context continue to hinder the identification of reliable therapeutic nodes (247). Finally, despite the rapid progress of single-cell and integrative multi-omics technologies, existing computational models remain inadequate for reconstructing the spatial and temporal trajectories of metastatic evolution. Bridging these gaps will require integrative frameworks that combine experimental biology with predictive modeling to map organ-specific colonization and therapeutic vulnerabilities (150, 246, 247).

### 5.2 Therapeutic limitations

A major limitation of the treatment of metastatic cancer is the lack of specificity of systemic therapies. Most systemic treatments are based on the molecular biology of primary tumors and do not adequately account for the distinct molecular and microenvironmental features of metastatic lesions. Consequently, these therapies often fail to achieve optimal efficacy at metastatic sites, which can vary substantially from the primary tumor in terms of gene expression profiles, immune landscape, and stromal composition. This lack of precision contributes to therapeutic failure in patients with advanced disease (13).

Off-target toxicity is another significant drawback of the conventional targeted therapies. Cytotoxic agents are effective in rapidly dividing cells and often damage healthy tissues, leading to adverse effects, such as myelosuppression, peripheral neuropathy, gastrointestinal disturbances, and generalized fatigue. These side effects can limit both the intensity and duration of treatment and substantially impact the patients’ quality of life. More selective agents, such as immunotherapies, can induce irAEs due to systemic immune activation, highlighting the need for more refined targeting strategies (248).

Additionally, microenvironmental barriers impede therapeutic efficacy in certain metastatic niches. Metastases frequently reside in organs, such as the brain, liver, and bone, with specialized physical and biochemical barriers. For instance, the blood–brain barrier restricts the entry of many therapeutic agents, whereas the bone stroma provides survival signals that promote resistance. Large biologics, such as monoclonal antibodies, face additional delivery challenges because less than 20% of the administered dose may reach the tumor site owing to the size and physical properties of the tumor interstitium (249).

Drug resistance remains a major obstacle to the management of metastatic diseases. Metastatic cancer cells exhibit substantial genetic and epigenetic plasticity, enabling them to rapidly evolve resistance mechanisms under therapeutic pressure. Common resistance mechanisms include mutations in drug targets, activation of compensatory or bypass pathways, and upregulation of efflux pumps. The inherent heterogeneity within and between tumor sites further complicates the application of uniform therapeutic regimens (250). As detailed earlier, organ-specific microenvironments not only restrict drug delivery but also actively drive therapeutic resistance through stromal signaling and adaptive survival mechanisms. These interactions blur the boundary between pharmacologic failure and biological resilience, underscoring the need for therapies that target both tumor cells and their supportive niches (251).

Metastatic tumors are particularly adept at immune evasion and employ several strategies to avoid detection and elimination. These include the upregulation of immune checkpoint molecules such as PD-L1, recruitment of immunosuppressive cell types such as regulatory T cells and myeloid-derived suppressor cells, and secretion of immunosuppressive cytokines. These mechanisms permit immune escape and reduce the effectiveness of immunotherapies that rely on intact antitumor immune responses (252).

Another limitation lies in the narrow focus of current therapies, many of which are primarily designed to target primary tumors. Few drugs have been specifically engineered to interfere with the unique biology of the metastatic cascade, including its dissemination, colonization, and outgrowth. This historical bias toward primary tumors has hampered the development of agents that can directly prevent or treat metastases (169).

The lack of reliable metastasis-specific biomarkers presents a further barrier to treatment optimization. Without molecular markers to predict metastatic potential or therapeutic response, clinicians have limited ability to personalize therapy or adapt treatment strategies over time. This issue is exacerbated by the fact that standard imaging modalities and biopsy approaches may not capture the full extent of tumor heterogeneity (253).

Preclinical models remain inadequate for studying metastasis. Most fail to capture the complexity of tumor–microenvironment interactions. This gap limits drug translation and slows progress in developing effective therapies.

Despite these challenges, it is crucial to recognize the Anti-Metastatic Paradox: Why Most Drugs Fail Clinical Translation. Although over 90% of anti-metastatic drugs have failed in clinical trials, this reflects deep biological complexity, preclinical limitations, and translational barriers. Tumor plasticity alongside redundant signaling pathways enables cancer cells to bypass single-target therapies, fostering drug resistance and recurrence. Preclinical models often fail to recapitulate human metastatic microenvironments, especially the physical and biochemical barriers unique to organs like bone and brain, which lead to overestimation of drug efficacy. Furthermore, inadequate drug delivery to metastatic niches occurs due to poor penetration and distribution, compounded by variability in nanoparticle biodistribution. Clinical trial designs suffer from insufficient biomarker-guided patient stratification, frequently relying on protein expression rather than pathway activity, resulting in diluted therapeutic signals across heterogeneous metastatic populations. Collectively, these factors underscore the urgent need for multi-targeted approaches, physiologically relevant models, precision biomarker strategies, and improved nanomedicine design to advance effective anti-metastatic therapies (254–256).

### 5.3 The potential of interdisciplinary collaboration

Interdisciplinary collaboration is essential for overcoming the systemic barriers that have hindered progress in metastatic cancer therapy. Integrating nanotechnology, immunology, and computational biology enables the translation of complex biological insights such as metastatic niche signaling and drug resistance into engineered therapeutic and diagnostic solutions. This convergence of disciplines is not merely additive but synergistic, transforming metastasis research from descriptive biology into predictive and intervention oriented science (257).

#### 5.3.1 Integrating nanotechnology and immunology

Nanotechnology provides the structural framework for delivering therapeutics with metastatic precision by enhancing localization, minimizing systemic toxicity, and enabling simultaneous imaging and treatment. Functionalized nanoparticles such as liposomes, micelles, dendrimers, and carbon nanotubes improve drug accumulation in metastatic lesions and permit real-time visualization of tumor burden. Clinical examples include liposomal doxorubicin for metastatic ovarian cancer and paclitaxel-loaded micelles for breast cancer (258, 259).

Immunotherapy, particularly immune checkpoint blockade and CAR T-cell therapy, has transformed the treatment landscape of advanced cancers yet remains constrained by immune evasion and suppressive microenvironments. Nanoparticle-assisted immunotherapy directly addresses these limitations by delivering immunostimulatory payloads to metastatic niches, reprogramming local immune cells, and sustaining cytotoxic responses. Hybrid nanostructures functioning as artificial antigen-presenting cells or co-carriers of chemotherapeutics and immune modulators exemplify multimodal designs that both attack tumors and recalibrate immunity (260).

More integrated approaches are emerging in the form of theranostic nanoparticles, which unite therapeutic and diagnostic functions to enable real-time monitoring of treatment response. Technologies such as photothermal and hyperthermic systems using gold nanoparticles or carbon nanotubes demonstrate how interdisciplinary design translates biological understanding into spatially controlled tumor eradication. **Table 1** summarizes these cross-disciplinary strategies and their relevance to metastatic control.

**Table 1:** Interdisciplinary innovations in metastatic cancer therapy.

Approach	Description	Example application
Targeted drug delivery	Nanoparticles deliver therapeutic agents specifically to metastatic tumor sites, reducing systemic toxicity.	Chitosan-based nanoparticles co-delivering siRNA against Snail and Twist transcription factors alongside paclitaxel effectively reversed EMT and suppressed lung metastases in breast cancer models (261).
Aging and theranostics	Nanoparticles integrate imaging and therapy to facilitate real-time tracking of metastasis-targeted intervention.	Hypoxia-responsive gold nanoparticles conjugated with nitroimidazole enable both photothermal ablation and imaging of hypoxic metastatic tumors (262).
Immune activation	Nanocarriers deliver immunostimulatory agents to remodel the premetastatic niche and enhance anti-tumor immunity.	Cell membrane-coated magnetic nanoparticles (gCM-MNs) stimulate macrophage repolarization (M2→M1) and block CD47–SIRPα signaling, reducing metastatic spread in melanoma and breast cancer (263).
Artificial APCs	Engineered biomimetic nanostructures replicate antigen presentation to boost immune-cell cross-talk.	Synthetic antigen-presenting nanoparticles loaded with neoantigen peptides improved T-cell infiltration into ECM-dense metastatic niches, enhancing CAR-T efficacy (100, 264).
Combination therapeutics	Co-delivery of multiple therapeutic agents using nanoplatforms to target synergistic metastatic pathways.	Dual-delivery nanoparticles combining doxorubicin with MMP inhibitors suppressed ECM remodeling, improved T-cell tumor infiltration, and limited metastasis formation (98, 265).
Hypoxia-directed strategies	Nanomaterials targeting tumor hypoxia to overcome resistance mechanisms promoting metastasis.	Perfluorocarbon-based oxygen-shuttling nanocarriers and carbonic anhydrase IX-targeting nanoparticles reversed drug resistance in hypoxic renal carcinoma models (116, 266, 267).

#### 5.3.2 Broader interdisciplinary approaches

Beyond nanotechnology and immunology, interdisciplinary collaboration among molecular biologists, bioengineers, computational scientists, and clinicians is redefining how metastasis is studied and treated. Integrating computational modeling with experimental biology enables dynamic prediction of metastatic spread, therapeutic resistance, and treatment response. Bioengineers contribute by designing adaptive delivery systems and implantable devices that translate these predictive insights into controlled, site-specific interventions. Together, these efforts create a feedback loop between data and therapy, accelerating the translation of mechanistic discoveries into clinically actionable strategies (268).

## 6 Conclusion

Cancer invasion and metastasis remain the leading causes of cancer mortality, driven by the interplay between cellular plasticity, microenvironmental remodeling, and systemic adaptation. Despite major advances in understanding the molecular mechanisms that govern these processes, effective translation into durable clinical therapies remains limited. Tumor heterogeneity, therapy resistance, microenvironmental protection, and the absence of experimental systems that fully recapitulate metastatic complexity continue to hinder therapeutic success.

Emerging technologies are beginning to bridge these gaps by linking mechanistic discovery to clinical application. Integrated approaches that combine multi-omics and single-cell analyses with nanotechnology-based delivery systems and CRISPR-mediated gene editing are redefining how metastasis can be detected, monitored, and treated. These innovations enable real-time tracking of metastatic evolution, precision targeting of disseminated cells, and rational design of combination therapies that counter both cellular and microenvironmental resistance mechanisms.

Progress now depends on advancing predictive modeling, validating metastasis-specific biomarkers, and designing adaptive therapeutic strategies that reflect the dynamic and heterogeneous nature of metastatic disease. Achieving this will require close interdisciplinary collaboration among molecular biologists, clinicians, engineers, and computational scientists, supported by inclusive and well-stratified clinical research. Through such integration, metastasis research can shift from descriptive biology to actionable, mechanism-driven interventions that transform patient outcomes.

## References

[1] Guan X . Cancer metastases: challenges and opportunities. Acta Pharm Sin B. (2015) 5:402–18. doi: 10.1016/j.apsb.2015.07.005, PMID: 26579471 PMC4629446

[2] Martin TA Ye L Sanders AJ Lane J Jiang WG . Cancer invasion and metastasis: molecular and cellular perspective. Madame Curie Bioscience Database [Internet]: Landes Bioscience. (2013).

[3] Lazebnik Y . What are the hallmarks of cancer? Nat Rev Cancer. (2010) 10:232–3. 10.1038/nrc2827 20355252

[4] Jiang WG Sanders AJ Katoh M Ungefroren H Gieseler F Prince M . Tissue invasion and metastasis: Molecular, biological and clinical perspectives. Semin Cancer Biol. (2015) 35 Suppl:S244–s75. doi: 10.1016/j.semcancer.2015.03.008, PMID: 25865774

[5] Budczies J von Winterfeld M Klauschen F Bockmayr M Lennerz JK Denkert C . The landscape of metastatic progression patterns across major human cancers. Oncotarget. (2014) 6:570. doi: 10.18632/oncotarget.2677, PMID: 25402435 PMC4381616

[6] Asif PJ Longobardi C Hahne M Medema JP . The role of cancer-associated fibroblasts in cancer invasion and metastasis. Cancers. (2021) 13:4720. doi: 10.3390/cancers13184720, PMID: 34572947 PMC8472587

[7] Leber MF Efferth T . Molecular principles of cancer invasion and metastasis. Int J Oncol. (2009) 34:881–95. 10.3892/ijo_00000214 19287945

[8] Chaffer CL Weinberg RA . A perspective on cancer cell metastasis. science. (2011) 331:1559–64. doi: 10.1126/science.1203543, PMID: 21436443

[9] Tahtamouni L Ahram M Koblinski J Rolfo C . Molecular regulation of cancer cell migration, invasion, and metastasis. Analytical Cell Pathol (Amsterdam). (2019) 2019:1356508. doi: 10.1155/2019/1356508, PMID: 31218208 PMC6536998

[10] Kwon MJ . Matrix metalloproteinases as therapeutic targets in breast cancer. Front Oncol. (2023) 12. doi: 10.3389/fonc.2022.1108695, PMID: 36741729 PMC9897057

[11] He DN Wang N Wen XL Li XH Guo Y Fu SH . Multi-omics analysis reveals a molecular landscape of the early recurrence and early metastasis in pan-cancer. Front Genet. (2023) 14:1061364. doi: 10.3389/fgene.2023.1061364, PMID: 37152984 PMC10157260

[12] Golas MM Gunawan B Gutenberg A Danner BC Gerdes JS Stadelmann C . Cytogenetic signatures favoring metastatic organotropism in colorectal cancer. Nat Commun. (2025) 16:3261. doi: 10.1038/s41467-025-58413-1, PMID: 40188208 PMC11972295

[13] Ganesh K Massagué J . Targeting metastatic cancer. Nat Med. (2021) 27:34–44. doi: 10.1038/s41591-020-01195-4, PMID: 33442008 PMC7895475

[14] Maiques O Fanshawe B Crosas-Molist E Rodriguez-Hernandez I Volpe A Cantelli G . A preclinical pipeline to evaluate migrastatics as therapeutic agents in metastatic melanoma. Br J Cancer. (2021) 125:699–713. doi: 10.1038/s41416-021-01442-6, PMID: 34172930 PMC8405734

[15] Liu F Zhang XH . Decoding metastatic microenvironments through single-cell omics reveals new insights into niche dynamics and tumor evolution. PloS Biol. (2025) 23:e3003299. doi: 10.1371/journal.pbio.3003299, PMID: 40658744 PMC12273945

[16] Wang C Li J Chen J Wang Z Zhu G Song L . Multi-omics analyses reveal biological and clinical insights in recurrent stage I non-small cell lung cancer. Nat Commun. (2025) 16:1477. doi: 10.1038/s41467-024-55068-2, PMID: 39929832 PMC11811181

[17] Shi Z-D Pang K Wu Z-X Dong Y Hao L Qin J-X . Tumor cell plasticity in targeted therapy-induced resistance: mechanisms and new strategies. Signal Transduction Targeted Ther. (2023) 8:113. doi: 10.1038/s41392-023-01383-x, PMID: 36906600 PMC10008648

[18] McGranahan N Swanton C . Clonal Heterogeneity and Tumor Evolution: Past, Present, and the Future. Cell. (2017) 168:613–28. doi: 10.1016/j.cell.2017.01.018, PMID: 28187284

[19] Dagogo-Jack I Shaw AT . Tumour heterogeneity and resistance to cancer therapies. Nat Rev Clin Oncol. (2018) 15:81–94. doi: 10.1038/nrclinonc.2017.166, PMID: 29115304

[20] Kuo HY Khan KA Kerbel RS . Antiangiogenic-immune-checkpoint inhibitor combinations: lessons from phase III clinical trials. Nat Rev Clin Oncol. (2024) 21:468–82. doi: 10.1038/s41571-024-00886-y, PMID: 38600370

[21] Mason WP . End of the road: confounding results of the CORE trial terminate the arduous journey of cilengitide for glioblastoma. Neuro Oncol. (2015) 17:634–5. doi: 10.1093/neuonc/nov018, PMID: 25681307 PMC4482863

[22] Thiery JP Acloque H Huang RYJ Nieto MA . Epithelial-Mesenchymal Transitions in Development and Disease. Cell. (2009) 139:871–90. doi: 10.1016/j.cell.2009.11.007, PMID: 19945376

[23] Brabletz S Schuhwerk H Brabletz T Stemmler MP . Dynamic EMT: a multi-tool for tumor progression. EMBO J. (2021) 40:e108647. doi: 10.15252/embj.2021108647, PMID: 34459003 PMC8441439

[24] Nieto MA Huang RY Jackson RA Thiery JP . EMT: 2016. Cell. (2016) 166:21–45. doi: 10.1016/j.cell.2016.06.028, PMID: 27368099

[25] Cano A Pérez-Moreno MA Rodrigo I Locascio A Blanco MJ del Barrio MG . The transcription factor snail controls epithelial-mesenchymal transitions by repressing E-cadherin expression. Nat Cell Biol. (2000) 2:76–83. doi: 10.1038/35000025, PMID: 10655586

[26] Yang J Mani SA Donaher JL Ramaswamy S Itzykson RA Come C . Twist, a Master Regulator of Morphogenesis, Plays an Essential Role in Tumor Metastasis. Cell. (2004) 117:927–39. doi: 10.1016/j.cell.2004.06.006, PMID: 15210113

[27] Krebs AM Mitschke J Lasierra Losada M Schmalhofer O Boerries M Busch H . The EMT-activator Zeb1 is a key factor for cell plasticity and promotes metastasis in pancreatic cancer. Nat Cell Biol. (2017) 19:518–29. doi: 10.1038/ncb3513, PMID: 28414315

[28] Brabletz T Jung A Spaderna S Hlubek F Kirchner T . Opinion: migrating cancer stem cells - an integrated concept of malignant tumour progression. Nat Rev Cancer. (2005) 5:744–9. doi: 10.1038/nrc1694, PMID: 16148886

[29] Chaffer CL Brennan JP Slavin JL Blick T Thompson EW Williams ED . Mesenchymal-to-Epithelial Transition Facilitates Bladder Cancer Metastasis: Role of Fibroblast Growth Factor Receptor-2. Cancer Res. (2006) 66:11271–8. doi: 10.1158/0008-5472.CAN-06-2044, PMID: 17145872

[30] Yao D Dai C Peng S . Mechanism of the Mesenchymal–Epithelial Transition and Its Relationship with Metastatic Tumor Formation. Mol Cancer Res. (2011) 9:1608–20. doi: 10.1158/1541-7786.MCR-10-0568, PMID: 21840933

[31] Ocaña OH Córcoles R Fabra A Moreno-Bueno G Acloque H Vega S . Metastatic colonization requires the repression of the epithelial-mesenchymal transition inducer Prrx1. Cancer Cell. (2012) 22:709–24. doi: 10.1016/j.ccr.2012.10.012, PMID: 23201163

[32] Thiery JP . Epithelial-mesenchymal transitions in tumour progression. Nat Rev Cancer. (2002) 2:442–54. doi: 10.1038/nrc822, PMID: 12189386

[33] Tsai JH Donaher JL Murphy DA Chau S Yang J . Spatiotemporal regulation of epithelial-mesenchymal transition is essential for squamous cell carcinoma metastasis. Cancer Cell. (2012) 22:725–36. doi: 10.1016/j.ccr.2012.09.022, PMID: 23201165 PMC3522773

[34] Del Pozo Martin Y Park D Ramachandran A Ombrato L Calvo F Chakravarty P . Mesenchymal Cancer Cell-Stroma Crosstalk Promotes Niche Activation, Epithelial Reversion, and Metastatic Colonization. Cell Rep. (2015) 13:2456–69. doi: 10.1016/j.celrep.2015.11.025, PMID: 26670048 PMC4695340

[35] Esposito M Mondal N Greco TM Wei Y Spadazzi C Lin SC . Bone vascular niche E-selectin induces mesenchymal-epithelial transition and Wnt activation in cancer cells to promote bone metastasis. Nat Cell Biol. (2019) 21:627–39. doi: 10.1038/s41556-019-0309-2, PMID: 30988423 PMC6556210

[36] Youssef KK Nieto MA . Epithelial-mesenchymal transition in tissue repair and degeneration. Nat Rev Mol Cell Biol. (2024) 25:720–39. doi: 10.1038/s41580-024-00733-z, PMID: 38684869

[37] Yang J Antin P Berx G Blanpain C Brabletz T Bronner M . Guidelines and definitions for research on epithelial-mesenchymal transition. Nat Rev Mol Cell Biol. (2020) 21:341–52. doi: 10.1038/s41580-020-0237-9, PMID: 32300252 PMC7250738

[38] Grande MT Sánchez-Laorden B López-Blau C De Frutos CA Boutet A Arévalo M . Snail1-induced partial epithelial-to-mesenchymal transition drives renal fibrosis in mice and can be targeted to reverse established disease. Nat Med. (2015) 21:989–97. doi: 10.1038/nm.3901, PMID: 26236989

[39] Lovisa S LeBleu VS Tampe B Sugimoto H Vadnagara K Carstens JL . Epithelial-to-mesenchymal transition induces cell cycle arrest and parenchymal damage in renal fibrosis. Nat Med. (2015) 21:998–1009. doi: 10.1038/nm.3902, PMID: 26236991 PMC4587560

[40] Pastushenko I Brisebarre A Sifrim A Fioramonti M Revenco T Boumahdi S . Identification of the tumour transition states occurring during EMT. Nature. (2018) 556:463–8. doi: 10.1038/s41586-018-0040-3, PMID: 29670281

[41] Kröger C Afeyan A Mraz J Eaton EN Reinhardt F Khodor YL . Acquisition of a hybrid E/M state is essential for tumorigenicity of basal breast cancer cells. Proc Natl Acad Sci U S A. (2019) 116:7353–62. doi: 10.1073/pnas.1812876116, PMID: 30910979 PMC6462070

[42] Simeonov KP Byrns CN Clark ML Norgard RJ Martin B Stanger BZ . Single-cell lineage tracing of metastatic cancer reveals selection of hybrid EMT states. Cancer Cell. (2021) 39:1150–62.e9. doi: 10.1016/j.ccell.2021.05.005, PMID: 34115987 PMC8782207

[43] Bracken CP Goodall GJ . The many regulators of epithelial–mesenchymal transition. Nat Rev Mol Cell Biol. (2022) 23:89–90. doi: 10.1038/s41580-021-00442-x, PMID: 34887545

[44] Tran HD Luitel K Kim M Zhang K Longmore GD Tran DD . Transient SNAIL1 expression is necessary for metastatic competence in breast cancer. Cancer Res. (2014) 74:6330–40. doi: 10.1158/0008-5472.CAN-14-0923, PMID: 25164016 PMC4925010

[45] Reichert M Bakir B Moreira L Pitarresi JR Feldmann K Simon L . Regulation of Epithelial Plasticity Determines Metastatic Organotropism in Pancreatic Cancer. Dev Cell. (2018) 45:696–711.e8. doi: 10.1016/j.devcel.2018.05.025, PMID: 29920275 PMC6011231

[46] Deng X Terunuma H . Harnessing NK Cells to Control Metastasis. Vaccines. (2022) 10:2018. doi: 10.3390/vaccines10122018, PMID: 36560427 PMC9781233

[47] Zimmermann RC Welch DR . BRMS1: a multifunctional signaling molecule in metastasis. Cancer Metastasis Rev. (2020) 39:755–68. doi: 10.1007/s10555-020-09871-0, PMID: 32232621 PMC7487056

[48] Al-Khater KM Almofty S Ravinayagam V Alrushaid N Rehman S . Role of a metastatic suppressor gene KAI1/CD82 in the diagnosis and prognosis of breast cancer. Saudi J Biol Sci. (2021) 28:3391–8. doi: 10.1016/j.sjbs.2021.03.001, PMID: 34121877 PMC8176039

[49] Min H-Y Lee H-Y . Cellular Dormancy in Cancer: Mechanisms and Potential Targeting Strategies. Cancer Res Treat. (2023) 55:720–36. doi: 10.4143/crt.2023.468, PMID: 36960624 PMC10372609

[50] Bae SY Kamalanathan KJ Galeano-Garces C Konety BR Antonarakis ES Parthasarathy J . Dissemination of Circulating Tumor Cells in Breast and Prostate Cancer: Implications for Early Detection. Endocrinology. (2024) 165. doi: 10.1210/endocr/bqae022, PMID: 38366552 PMC10904107

[51] Lu Y Lian S Cheng Y Ye Y Xie X Fu C . Circulation patterns and seed-soil compatibility factors cooperate to cause cancer organ-specific metastasis. Exp Cell Res. (2019) 375:62–72. doi: 10.1016/j.yexcr.2018.12.015, PMID: 30578764

[52] Massagué J Obenauf AC . Metastatic colonization by circulating tumour cells. Nature. (2016) 529:298–306. doi: 10.1038/nature17038, PMID: 26791720 PMC5029466

[53] Takanami I . Overexpression of CCR7 mRNA in nonsmall cell lung cancer: correlation with lymph node metastasis. Int J Cancer. (2003) 105:186–9. doi: 10.1002/ijc.11063, PMID: 12673677

[54] Ding Y Shimada Y Maeda M Kawabe A Kaganoi J Komoto I . Association of CC chemokine receptor 7 with lymph node metastasis of esophageal squamous cell carcinoma1. Clin Cancer Res. (2003) 9:3406–12., PMID: 12960129

[55] Mashino K Sadanaga N Yamaguchi H Tanaka F Ohta M Shibuta K . Expression of chemokine receptor CCR7 is associated with lymph node metastasis of gastric carcinoma1. Cancer Res. (2002) 62:2937–41., PMID: 12019175

[56] Teicher BA Fricker SP . CXCL12 (SDF-1)/CXCR4 pathway in cancer. Clin Cancer Res. (2010) 16:2927–31. doi: 10.1158/1078-0432.CCR-09-2329, PMID: 20484021

[57] Zhan Q Liu B Situ X Luo Y Fu T Wang Y . New insights into the correlations between circulating tumor cells and target organ metastasis. Signal Transduction Targeted Ther. (2023) 8:465. doi: 10.1038/s41392-023-01725-9, PMID: 38129401 PMC10739776

[58] Zeng Z Li Y Pan Y Lan X Song F Sun J . Cancer-derived exosomal miR-25-3p promotes pre-metastatic niche formation by inducing vascular permeability and angiogenesis. Nat Commun. (2018) 9:5395. doi: 10.1038/s41467-018-07810-w, PMID: 30568162 PMC6300604

[59] Wang Y Jia J Wang F Fang Y Yang Y Zhou Q . Pre-metastatic niche: formation, characteristics and therapeutic implication. Signal Transduction Targeted Ther. (2024) 9:236. doi: 10.1038/s41392-024-01937-7, PMID: 39317708 PMC11422510

[60] Gao Y Rosen JM Zhang XH-F . The tumor-immune ecosystem in shaping metastasis. Am J Physiology-Cell Physiol. (2023) 324:C707–17. doi: 10.1152/ajpcell.00132.2022, PMID: 36717100 PMC10027084

[61] Carrolo M Miranda JAI Vilhais G Quintela A Sousa MFE Costa DA . Metastatic organotropism: a brief overview. Front Oncol. (2024) 14:1358786. doi: 10.3389/fonc.2024.1358786, PMID: 38725618 PMC11079203

[62] Kaszak I Witkowska-Piłaszewicz O Niewiadomska Z Dworecka-Kaszak B Ngosa Toka F Jurka P . Role of cadherins in cancer—A review. Int J Mol Sci. (2020) 21:7624. doi: 10.3390/ijms21207624, PMID: 33076339 PMC7589192

[63] Cáceres-Calle D Torre-Cea I Marcos-Zazo L Carrera-Aguado I Guerra-Paes E Berlana-Galán P . Integrins as key mediators of metastasis. Int J Mol Sci. (2025) 26:904. doi: 10.3390/ijms26030904, PMID: 39940673 PMC11816423

[64] Natoni A Macauley MS O’Dwyer ME . Targeting selectins and their ligands in cancer. Front Oncol. (2016) 6: 2016. doi: 10.3389/fonc.2016.00093, PMID: 27148485 PMC4834419

[65] Mustafa S Koran S AlOmair L . Insights into the role of matrix metalloproteinases in cancer and its various therapeutic aspects: A review. Front Mol Biosci. (2022) 9:2022. doi: 10.3389/fmolb.2022.896099, PMID: 36250005 PMC9557123

[66] Wright K Ly T Kriet M Czirok A Thomas SM . Cancer-associated fibroblasts: master tumor microenvironment modifiers. Cancers (Basel). (2023) 15. doi: 10.3390/cancers15061899, PMID: 36980785 PMC10047485

[67] Schober M Jesenofsky R Faissner R Weidenauer C Hagmann W Michl P . Desmoplasia and chemoresistance in pancreatic cancer. Cancers. (2014) 6:2137–54. doi: 10.3390/cancers6042137, PMID: 25337831 PMC4276960

[68] Lee HH Al-Ogaili Z . Fibroblast activation protein and the tumour microenvironment: challenges and therapeutic opportunities. Oncol Rev. (2025) 19:2025. doi: 10.3389/or.2025.1617487, PMID: 40741380 PMC12308159

[69] Feng X Cao F Wu X Xie W Wang P Jiang H . Targeting extracellular matrix stiffness for cancer therapy. Frontiers in Immunology. (2024) 15:2024. doi: 10.3389/fimmu.2024.1467602, PMID: 39697341 PMC11653020

[70] Chen H Chen J Yuan H Li X Li W . Hypoxia−inducible factor−1α: A critical target for inhibiting the metastasis of hepatocellular carcinoma. Oncol Lett. (2022) 24:1–9. doi: 10.3892/ol.2022.13404, PMID: 35814827 PMC9260738

[71] Tam SY Wu VWC Law HKW . Hypoxia-induced epithelial-mesenchymal transition in cancers: HIF-1α and beyond. Front Oncol. (2020) 10:486. doi: 10.3389/fonc.2020.00486, PMID: 32322559 PMC7156534

[72] Tang M Bolderson E O’Byrne KJ Richard DJ . Tumor hypoxia drives genomic instability. Frontiers in cell and developmental biology. (2021) 9:2021. doi: 10.3389/fcell.2021.626229, PMID: 33796526 PMC8007910

[73] Leong SP Witte MH . Cancer metastasis through the lymphatic versus blood vessels. Clin Exp Metastasis. (2024) 41:387–402. doi: 10.1007/s10585-024-10288-0, PMID: 38940900 PMC11374872

[74] Krakhmal NV Zavyalova M Denisov E Vtorushin S Perelmuter V . Cancer invasion: patterns and mechanisms. Acta Naturae. (2015) 7:17–28. doi: 10.32607/20758251-2015-7-2-17-28 26085941 PMC4463409

[75] Wu J-s Jiang J Chen B-j Wang K Tang Y-l Liang X-h . Plasticity of cancer cell invasion: Patterns and mechanisms. Trans Oncol. (2021) 14:100899. doi: 10.1016/j.tranon.2020.100899, PMID: 33080522 PMC7573380

[76] Alonso-Matilla R Provenzano PP Odde DJ . Physical principles and mechanisms of cell migration. NPJ Biol Phys Mechanics. (2025) 2:2. doi: 10.1038/s44341-024-00008-w, PMID: 39829952 PMC11738987

[77] Jiang M Fang H Tian H . Metabolism of cancer cells and immune cells in the initiation, progression, and metastasis of cancer. Theranostics. (2025) 15:155. doi: 10.7150/thno.103376, PMID: 39744225 PMC11667227

[78] Janssen LM Ramsay EE Logsdon CD Overwijk WW . The immune system in cancer metastasis: friend or foe? J Immunotherapy Cancer. (2017) 5:1–14. 10.1186/s40425-017-0283-9 PMC5644253 29037250

[79] El-Kenawi A Hänggi K Ruffell B . The immune microenvironment and cancer metastasis. Cold Spring Harbor Perspect Med. (2020) 10:a037424. doi: 10.1101/cshperspect.a037424, PMID: 31501262 PMC7117953

[80] Gewalt T Diehl L Meder L . Tumor and immune cell interactions in the formation of organ-specific metastasis. Front Oncol. (2024) 14:1373308. doi: 10.3389/fonc.2024.1373308, PMID: 38444685 PMC10914249

[81] Ardiani A Gameiro SR Palena C Hamilton DH Kwilas A King TH . Vaccine-mediated immunotherapy directed against a transcription factor driving the metastatic process. Cancer Res. (2014) 74:1945–57. doi: 10.1158/0008-5472.CAN-13-2045, PMID: 24520078 PMC4697465

[82] Schacke M Kumar J Colwell N Hermanson K Folle GA Nechaev S . PARP-1/2 inhibitor olaparib prevents or partially reverts EMT induced by TGF-β in NMuMG cells. Int J Mol Sci. (2019) 20:518. doi: 10.3390/ijms20030518, PMID: 30691122 PMC6387051

[83] Yang T Chen M Sun T . Simvastatin attenuates TGF-β1-induced epithelial-mesenchymal transition in human alveolar epithelial cells. Cell Physiol Biochem. (2013) 31:863–74. doi: 10.1159/000350104, PMID: 23817018

[84] Lustberg MB Pant S Ruppert AS Shen T Wei Y Chen L . Phase I/II trial of non-cytotoxic suramin in combination with weekly paclitaxel in metastatic breast cancer treated with prior taxanes. Cancer Chemotherapy Pharmacol. (2012) 70:49–56. doi: 10.1007/s00280-012-1887-x, PMID: 22729159 PMC3466596

[85] Chen B Dragomir MP Yang C Li Q Horst D Calin GA . Targeting non-coding RNAs to overcome cancer therapy resistance. Signal Transduction Targeted Ther. (2022) 7:121. doi: 10.1038/s41392-022-00975-3, PMID: 35418578 PMC9008121

[86] Lippert TH Ruoff H-J Volm M . Intrinsic and acquired drug resistance in Malignant tumors. Arzneimittelforschung. (2008) 58:261–4. 10.1055/s-0031-1296504 18677966

[87] Xu Q Deng F Qin Y Zhao Z Wu Z Xing Z . Long non-coding RNA regulation of epithelial–mesenchymal transition in cancer metastasis. Cell Death Dis. (2016) 7:e2254–4. doi: 10.1038/cddis.2016.149, PMID: 27277676 PMC5143379

[88] Korpal M Lee ES Hu G Kang Y . The miR-200 family inhibits epithelial-mesenchymal transition and cancer cell migration by direct targeting of E-cadherin transcriptional repressors ZEB1 and ZEB2. J Biol Chem. (2008) 283:14910–4. doi: 10.1074/jbc.C800074200, PMID: 18411277 PMC3258899

[89] Martinez-Iglesias O Casas-Pais A Castosa R Díaz-Díaz A Roca-Lema D Concha Á . Hakin-1, a new specific small-molecule inhibitor for the E3 ubiquitin-ligase Hakai, inhibits carcinoma growth and progression. Cancers. (2020) 12:1340. doi: 10.3390/cancers12051340, PMID: 32456234 PMC7281109

[90] Lambies G Miceli M Martínez-Guillamon C Olivera-Salguero R Peña R Frías C-P . TGFβ-activated USP27X deubiquitinase regulates cell migration and chemoresistance via stabilization of Snail1. Cancer Res. (2019) 79:33–46. doi: 10.1158/0008-5472.CAN-18-0753, PMID: 30341066 PMC9386731

[91] Li N Babaei-Jadidi R Lorenzi F Spencer-Dene B Clarke P Domingo E . An FBXW7-ZEB2 axis links EMT and tumour microenvironment to promote colorectal cancer stem cells and chemoresistance. Oncogenesis. (2019) 8:13. doi: 10.1038/s41389-019-0125-3, PMID: 30783098 PMC6381143

[92] Bangarh R Saini RV Saini AK Singh T Joshi H Ramniwas S . Dynamics of epithelial-mesenchymal plasticity driving cancer drug resistance. Cancer Pathogenesis Ther. (2025) 3:120–8. doi: 10.1016/j.cpt.2024.07.002, PMID: 40182126 PMC11963173

[93] Ramesh V Brabletz T Ceppi P . Targeting EMT in cancer with repurposed metabolic inhibitors. Trends Cancer. (2020) 6:942–50. doi: 10.1016/j.trecan.2020.06.005, PMID: 32680650

[94] Morera DS Hennig MS Talukder A Lokeshwar SD Wang J Garcia-Roig M . Hyaluronic acid family in bladder cancer: potential prognostic biomarkers and therapeutic targets. Br J Cancer. (2017) 117:1507–17. doi: 10.1038/bjc.2017.318, PMID: 28972965 PMC5680466

[95] Liu Y Mao C Wang M Liu N Ouyang L Liu S . Cancer progression is mediated by proline catabolism in non-small cell lung cancer. Oncogene. (2020) 39:2358–76. doi: 10.1038/s41388-019-1151-5, PMID: 31911619

[96] Wu X Li X Fu Q Cao Q Chen X Wang M . AKR1B1 promotes basal-like breast cancer progression by a positive feedback loop that activates the EMT program. J Exp Med. (2017) 214:1065–79. doi: 10.1084/jem.20160903, PMID: 28270406 PMC5379972

[97] Papadaki MA Stoupis G Theodoropoulos PA Mavroudis D Georgoulias V Agelaki S . Circulating tumor cells with stemness and epithelial-to-mesenchymal transition features are chemoresistant and predictive of poor outcome in metastatic breast cancer. Mol Cancer Ther. (2019) 18:437–47. doi: 10.1158/1535-7163.MCT-18-0584, PMID: 30401696

[98] Prakash J Shaked Y . The interplay between extracellular matrix remodeling and cancer therapeutics. Cancer Discov. (2024) 14:1375–88. doi: 10.1158/2159-8290.CD-24-0002, PMID: 39091205 PMC11294818

[99] Almutairi S Kalloush H Manoon NA Bardaweel SK . Matrix metalloproteinases inhibitors in cancer treatment: an updated review (2013–2023). Molecules. (2023) 28:5567. doi: 10.3390/molecules28145567, PMID: 37513440 PMC10384300

[100] Sleeboom JJF van Tienderen GS Schenke-Layland K van der Laan LJW Khalil AA Verstegen MMA . The extracellular matrix as hallmark of cancer and metastasis: From biomechanics to therapeutic targets. Sci Trans Med. (2024) 16:eadg3840. doi: 10.1126/scitranslmed.adg3840, PMID: 38170791

[101] Jabłońska-Trypuć A Matejczyk M Rosochacki S . Matrix metalloproteinases (MMPs), the main extracellular matrix (ECM) enzymes in collagen degradation, as a target for anticancer drugs. J Enzyme Inhibition Medicinal Chem. (2016) 31:177–83. doi: 10.3109/14756366.2016.1161620, PMID: 27028474

[102] Rasmussen HS McCann PP . Matrix metalloproteinase inhibition as a novel anticancer strategy: a review with special focus on batimastat and marimastat. Pharmacol Ther. (1997) 75:69–75. doi: 10.1016/S0163-7258(97)00023-5, PMID: 9364582

[103] Winer A Adams S Mignatti P . Matrix metalloproteinase inhibitors in cancer therapy: turning past failures into future successes. Mol Cancer Ther. (2018) 17:1147–55. doi: 10.1158/1535-7163.MCT-17-0646, PMID: 29735645 PMC5984693

[104] Murayama C Kawaguchi AT Kamijo A Naito K Iwao K Tsukamoto H . Liposome-encapsulated hemoglobin enhances chemotherapy to suppress metastasis in mice. Artif Organs. (2014) 38:656–61. doi: 10.1111/aor.12354, PMID: 25065266

[105] Deng Y Chakraborty P Jolly MK Levine H . A theoretical approach to coupling the epithelial-mesenchymal transition (EMT) to extracellular matrix (ECM) stiffness via LOXL2. Cancers. (2021) 13:1609. doi: 10.3390/cancers13071609, PMID: 33807227 PMC8037024

[106] Yuan Z Li Y Zhang S Wang X Dou H Yu X . Extracellular matrix remodeling in tumor progression and immune escape: from mechanisms to treatments. Mol Cancer. (2023) 22:48. doi: 10.1186/s12943-023-01744-8, PMID: 36906534 PMC10007858

[107] Semenza GL . Hypoxia-inducible factors in physiology and medicine. Cell. (2012) 148:399–408. doi: 10.1016/j.cell.2012.01.021, PMID: 22304911 PMC3437543

[108] Mustafa M Rashed M Winum J-Y . Novel anticancer drug discovery strategies targeting hypoxia-inducible factors. Expert Opin Drug Discov. (2025) 20:103–21. doi: 10.1080/17460441.2024.2442739, PMID: 39670847

[109] Wilson WR Hay MP . Targeting hypoxia in cancer therapy. Nat Rev Cancer. (2011) 11:393–410. doi: 10.1038/nrc3064, PMID: 21606941

[110] Rankin EB Nam J-M Giaccia AJ . Hypoxia: signaling the metastatic cascade. Trends Cancer. (2016) 2:295–304. doi: 10.1016/j.trecan.2016.05.006, PMID: 28741527 PMC5808868

[111] Palayoor ST Mitchell JB Cerna D DeGraff W John-Aryankalayil M Coleman CN . PX-478, an inhibitor of hypoxia-inducible factor-1α, enhances radiosensitivity of prostate carcinoma cells. Int J Cancer. (2008) 123:2430–7. doi: 10.1002/ijc.23807, PMID: 18729192 PMC4277812

[112] Zhang H Qian DZ Tan YS Lee K Gao P Ren YR . Digoxin and other cardiac glycosides inhibit HIF-1α synthesis and block tumor growth. Proc Natl Acad Sci. (2008) 105:19579–86. doi: 10.1073/pnas.0809763105, PMID: 19020076 PMC2604945

[113] Wong CC-L Zhang H Gilkes DM Chen J Wei H Chaturvedi P . Inhibitors of hypoxia-inducible factor 1 block breast cancer metastatic niche formation and lung metastasis. J Mol Med. (2012) 90:803–15. doi: 10.1007/s00109-011-0855-y, PMID: 22231744 PMC3437551

[114] Gilkes DM . Hypoxia and cancer metastasis. Springer (2019).

[115] Wohlrab C Vissers MCM Phillips E Morrin H Robinson BA Dachs GU . The association between ascorbate and the hypoxia-inducible factors in human renal cell carcinoma requires a functional von hippel-lindau protein. Front Oncol. (2018) 8:574. doi: 10.3389/fonc.2018.00574, PMID: 30555801 PMC6284050

[116] Debnath SK Debnath M Ghosh A Srivastava R Omri A . Targeting tumor hypoxia with nanoparticle-based therapies: challenges, opportunities, and clinical implications. Pharmaceuticals. (2024) 17:1389. doi: 10.3390/ph17101389, PMID: 39459028 PMC11510357

[117] Li Y Zhao L Li XF . Targeting hypoxia: hypoxia-activated prodrugs in cancer therapy. Front Oncol. (2021) 11:700407. doi: 10.3389/fonc.2021.700407, PMID: 34395270 PMC8358929

[118] Batenburg MCT van den Bongard H Kleynen CE Maarse W Witkamp A Ernst M . Assessing the effect of hyperbaric oxygen therapy in breast cancer patients with late radiation toxicity (HONEY trial): a trial protocol using a trial within a cohort design. Trials. (2020) 21:980. doi: 10.1186/s13063-020-04869-z, PMID: 33246494 PMC7694912

[119] Vito A El-Sayes N Mossman K . Hypoxia-driven immune escape in the tumor microenvironment. Cells. (2020) 9:992. doi: 10.3390/cells9040992, PMID: 32316260 PMC7227025

[120] Mohammad GH Vassileva V Acedo P Olde Damink SWM Malago M Dhar DK . Targeting pyruvate kinase M2 and lactate dehydrogenase A is an effective combination strategy for the treatment of pancreatic cancer. Cancers (Basel). (2019) 11. doi: 10.3390/cancers11091372, PMID: 31527446 PMC6770573

[121] Shigeta K Hasegawa M Hishiki T Naito Y Baba Y Mikami S . IDH2 stabilizes HIF-1α-induced metabolic reprogramming and promotes chemoresistance in urothelial cancer. EMBO J. (2023) 42:e110620. doi: 10.15252/embj.2022110620, PMID: 36637036 PMC9929641

[122] Gilkes DM Semenza GL Wirtz D . Hypoxia and the extracellular matrix: drivers of tumour metastasis. Nat Rev Cancer. (2014) 14:430–9. doi: 10.1038/nrc3726, PMID: 24827502 PMC4283800

[123] Hugo W Zaretsky JM Sun L Song C Moreno BH Hu-Lieskovan S . Genomic and transcriptomic features of response to anti-PD-1 therapy in metastatic melanoma. Cell. (2016) 165:35–44. doi: 10.1016/j.cell.2016.02.065, PMID: 26997480 PMC4808437

[124] Hatfield SM Kjaergaard J Lukashev D Schreiber TH Belikoff B Abbott R . Immunological mechanisms of the antitumor effects of supplemental oxygenation. Sci Transl Med. (2015) 7:277ra30. doi: 10.1126/scitranslmed.aaa1260, PMID: 25739764 PMC4641038

[125] Baginska J Viry E Berchem G Poli A Noman MZ van Moer K . Granzyme B degradation by autophagy decreases tumor cell susceptibility to natural killer-mediated lysis under hypoxia. Proc Natl Acad Sci U.S.A. (2013) 110:17450–5., PMID: 24101526 10.1073/pnas.1304790110 PMC3808626

[126] Liu S Wei W Wang J Chen T . Theranostic applications of selenium nanomedicines against lung cancer. J Nanobiotechnology. (2023) 21:96. doi: 10.1186/s12951-023-01825-2, PMID: 36935493 PMC10026460

[127] Murphy DA Cheng H Yang T Yan X Adjei IM . Reversing hypoxia with PLGA-encapsulated manganese dioxide nanoparticles improves natural killer cell response to tumor spheroids. Mol Pharm. (2021) 18:2935–46. doi: 10.1021/acs.molpharmaceut.1c00085, PMID: 34191525

[128] Busk M Overgaard J Horsman MR . Imaging of tumor hypoxia for radiotherapy: current status and future directions. Semin Nucl Med. (2020) 50:562–83. doi: 10.1053/j.semnuclmed.2020.05.003, PMID: 33059825

[129] Wang L Liu WQ Broussy S Han B Fang H . Recent advances of anti-angiogenic inhibitors targeting VEGF/VEGFR axis. Front Pharmacol. (2023) 14:1307860. doi: 10.3389/fphar.2023.1307860, PMID: 38239196 PMC10794590

[130] Chung AS Lee J Ferrara N . Targeting the tumour vasculature: insights from physiological angiogenesis. Nat Rev Cancer. (2010) 10:505–14. doi: 10.1038/nrc2868, PMID: 20574450

[131] Ghalehbandi S Yuzugulen J Pranjol MZI Pourgholami MH . The role of VEGF in cancer-induced angiogenesis and research progress of drugs targeting VEGF. Eur J Pharmacol. (2023) 949:175586. doi: 10.1016/j.ejphar.2023.175586, PMID: 36906141

[132] Majidpoor J Mortezaee K . Angiogenesis as a hallmark of solid tumors - clinical perspectives. Cell Oncol (Dordr). (2021) 44:715–37. doi: 10.1007/s13402-021-00602-3, PMID: 33835425 PMC12980750

[133] Liu Z-L Chen H-H Zheng L-L Sun L-P Shi L . Angiogenic signaling pathways and anti-angiogenic therapy for cancer. Signal Transduction Targeted Ther. (2023) 8:198. doi: 10.1038/s41392-023-01460-1, PMID: 37169756 PMC10175505

[134] Oguntade AS Al-Amodi F Alrumayh A Alobaida M Bwalya M . Anti-angiogenesis in cancer therapeutics: the magic bullet. J Egyptian Natl Cancer Institute. (2021) 33:15. doi: 10.1186/s43046-021-00072-6, PMID: 34212275 PMC13316910

[135] Risau W . Mechanisms of angiogenesis. Nature. (1997) 386:671–4. doi: 10.1038/386671a0, PMID: 9109485

[136] Patel SA Nilsson MB Le X Cascone T Jain RK Heymach JV . Molecular mechanisms and future implications of VEGF/VEGFR in cancer therapy. Clin Cancer Res. (2023) 29:30–9. doi: 10.1158/1078-0432.CCR-22-1366, PMID: 35969170 PMC10274152

[137] Wang H Yin Y Li W Zhao X Yu Y Zhu J . Over-expression of PDGFR-β promotes PDGF-induced proliferation, migration, and angiogenesis of EPCs through PI3K/Akt signaling pathway. PloS One. (2012) 7:e30503. doi: 10.1371/journal.pone.0030503, PMID: 22355314 PMC3280261

[138] Wang N Jain RK Batchelor TT . New directions in anti-angiogenic therapy for glioblastoma. Neurotherapeutics. (2017) 14:321–32. doi: 10.1007/s13311-016-0510-y, PMID: 28083806 PMC5398985

[139] Incio J Ligibel JA McManus DT Suboj P Jung K Kawaguchi K . Obesity promotes resistance to anti-VEGF therapy in breast cancer by up-regulating IL-6 and potentially FGF-2. Sci Transl Med. (2018) 10. doi: 10.1126/scitranslmed.aag0945, PMID: 29540614 PMC5936748

[140] Bokhari SMZ Hamar P . Vascular endothelial growth factor-D (VEGF-D): an angiogenesis bypass in Malignant tumors. Int J Mol Sci. (2023) 24:13317. doi: 10.3390/ijms241713317, PMID: 37686121 PMC10487419

[141] Tu J Liang H Li C Huang Y Wang Z Chen X . The application and research progress of anti-angiogenesis therapy in tumor immunotherapy. Front Immunol. (2023) 14:2023. doi: 10.3389/fimmu.2023.1198972, PMID: 37334350 PMC10272381

[142] Ratajska A Jankowska-Steifer E Czarnowska E Olkowski R Gula G Niderla-Bielińska J . Vasculogenesis and its cellular therapeutic applications. Cells Tissues Organs. (2016) 203:141–52. doi: 10.1159/000448551, PMID: 27654624

[143] Song S Ewald AJ Stallcup W Werb Z Bergers G . PDGFRbeta+ perivascular progenitor cells in tumours regulate pericyte differentiation and vascular survival. Nat Cell Biol. (2005) 7:870–9. doi: 10.1038/ncb1288, PMID: 16113679 PMC2771163

[144] Tamura R Tanaka T Akasaki Y Murayama Y Yoshida K Sasaki H . The role of vascular endothelial growth factor in the hypoxic and immunosuppressive tumor microenvironment: perspectives for therapeutic implications. Med Oncol. (2019) 37:2. doi: 10.1007/s12032-019-1329-2, PMID: 31713115

[145] Ribatti D Solimando AG Pezzella F . The anti-VEGF(R) drug discovery legacy: improving attrition rates by breaking the vicious cycle of angiogenesis in cancer. Cancers (Basel). (2021) 13. doi: 10.3390/cancers13143433, PMID: 34298648 PMC8304542

[146] Elebiyo TC Rotimi D Evbuomwan IO Maimako RF Iyobhebhe M Ojo OA . Reassessing vascular endothelial growth factor (VEGF) in anti-angiogenic cancer therapy. Cancer Treat Res Commun. (2022) 32:100620. doi: 10.1016/j.ctarc.2022.100620, PMID: 35964475

[147] Angara K Borin TF Arbab AS . Vascular mimicry: A novel neovascularization mechanism driving anti-angiogenic therapy (AAT) resistance in glioblastoma. Transl Oncol. (2017) 10:650–60. doi: 10.1016/j.tranon.2017.04.007, PMID: 28668763 PMC5496207

[148] Guo Z Jing X Sun X Sun S Yang Y Cao Y . Tumor angiogenesis and anti-angiogenic therapy. Chin Med J (Engl). (2024) 137:2043–51. doi: 10.1097/CM9.0000000000003231, PMID: 39051171 PMC11374217

[149] Zhang C Wang H Li X Jiang Y Sun G Yu H . Enhancing antitumor immunity: the role of immune checkpoint inhibitors, anti-angiogenic therapy, and macrophage reprogramming. Front Oncol. (2025) 15:2025. doi: 10.3389/fonc.2025.1526407, PMID: 40260303 PMC12009726

[150] Tang C Fu S Jin X Li W Xing F Duan B . Personalized tumor combination therapy optimization using the single-cell transcriptome. Genome Med. (2023) 15:105. doi: 10.1186/s13073-023-01256-6, PMID: 38041202 PMC10691165

[151] Yang M Cui M Sun Y Liu S Jiang W . Mechanisms, combination therapy, and biomarkers in cancer immunotherapy resistance. Cell Communication Signaling. (2024) 22:338. doi: 10.1186/s12964-024-01711-w, PMID: 38898505 PMC11186190

[152] Gion M Blancas I Cortez-Castedo P Cortés-Salgado A Marmé F Blanch S . Atezolizumab plus paclitaxel and bevacizumab as first-line treatment of advanced triple-negative breast cancer: the ATRACTIB phase 2 trial. Nat Med. (2025). doi: 10.1038/s41591-025-03734-3, PMID: 40467896 PMC12353800

[153] He H Zhou F . Efficacy and safety of anti-angiogenic drugs combined with chemotherapy in the treatment of platinum-sensitive/resistant ovarian cancer: a meta-analysis with trial sequential analysis of randomized controlled trials. Front Pharmacol. (2024) 15:1446403. doi: 10.3389/fphar.2024.1446403, PMID: 39640492 PMC11617189

[154] Abdallah M Voland R Decamp M Flickinger J Pacioles T Jamil M . Evaluation of anti-angiogenic therapy combined with immunotherapy and chemotherapy as a strategy to treat locally advanced and metastatic non-small-cell lung cancer. Cancers. (2024) 16:4207. doi: 10.3390/cancers16244207, PMID: 39766108 PMC11674749

[155] Zhang Y Zhang Z . The history and advances in cancer immunotherapy: understanding the characteristics of tumor-infiltrating immune cells and their therapeutic implications. Cell Mol Immunol. (2020) 17:807–21. doi: 10.1038/s41423-020-0488-6, PMID: 32612154 PMC7395159

[156] Zhang Y Liu Z . Insights into the mechanisms of immune-checkpoint inhibitors gained from spatiotemporal dynamics of the tumor microenvironment. Advanced Sci. (2025) 12:e080692. doi: 10.1002/advs.202508692, PMID: 40874420 PMC12462942

[157] Korman AJ Garrett-Thomson SC Lonberg N . The foundations of immune checkpoint blockade and the ipilimumab approval decennial. Nat Rev Drug Discov. (2022) 21:509–28. doi: 10.1038/s41573-021-00345-8, PMID: 34937915

[158] Bagchi S Yuan R Engleman EG . Immune checkpoint inhibitors for the treatment of cancer: clinical impact and mechanisms of response and resistance. Annu Rev Pathol. (2021) 16:223–49. doi: 10.1146/annurev-pathol-042020-042741, PMID: 33197221

[159] Aden D Zaheer S Sureka N Trisal M Chaurasia JK Zaheer S . Exploring immune checkpoint inhibitors: Focus on PD-1/PD-L1 axis and beyond. Pathol Res Pract. (2025) 269:155864. doi: 10.1016/j.prp.2025.155864, PMID: 40068282

[160] Arafat Hossain M . A comprehensive review of immune checkpoint inhibitors for cancer treatment. Int Immunopharmacol. (2024) 143:113365. doi: 10.1016/j.intimp.2024.113365, PMID: 39447408

[161] Wolchok JD Chiarion-Sileni V Gonzalez R Rutkowski P Grob JJ Cowey CL . Overall survival with combined nivolumab and ipilimumab in advanced melanoma. N Engl J Med. (2017) 377:1345–56. doi: 10.1056/NEJMoa1709684, PMID: 28889792 PMC5706778

[162] Younis A Gribben J . Immune checkpoint inhibitors: fundamental mechanisms, current status and future directions. Immuno. (2024) 4:186–210. doi: 10.3390/immuno4030013

[163] Alsaafeen BH Ali BR Elkord E . Resistance mechanisms to immune checkpoint inhibitors: updated insights. Mol Cancer. (2025) 24:20. doi: 10.1186/s12943-024-02212-7, PMID: 39815294 PMC11734352

[164] Kong Y Li J Zhao X Wu Y Chen L . CAR-T cell therapy: developments, challenges and expanded applications from cancer to autoimmunity. Front Immunol. (2025) 15:2024. doi: 10.3389/fimmu.2024.1519671, PMID: 39850899 PMC11754230

[165] Sirini C De Rossi L Moresco MA Casucci M . CAR T cells in solid tumors and metastasis: paving the way forward. Cancer Metastasis Rev. (2024) 43:1279–96. doi: 10.1007/s10555-024-10213-7, PMID: 39316265

[166] Esfahani K Roudaia L Buhlaiga N Del Rincon SV Papneja N Miller WH Jr . A review of cancer immunotherapy: from the past, to the present, to the future. Curr Oncol. (2020) 27:S87–s97. doi: 10.3747/co.27.5223, PMID: 32368178 PMC7194005

[167] Hadiloo K Taremi S Heidari M Esmaeilzadeh A . The CAR macrophage cells, a novel generation of chimeric antigen-based approach against solid tumors. biomark Res. (2023) 11:103. doi: 10.1186/s40364-023-00537-x, PMID: 38017494 PMC10685521

[168] Wang Z Li P Zeng X Guo J Zhang C Fan Z . CAR-T therapy dilemma and innovative design strategies for next generation. Cell Death Dis. (2025) 16:211. doi: 10.1038/s41419-025-07454-x, PMID: 40148310 PMC11950394

[169] Li Y Liu F Cai Q Deng L Ouyang Q Zhang XH . Invasion and metastasis in cancer: molecular insights and therapeutic targets. Signal Transduct Target Ther. (2025) 10:57. doi: 10.1038/s41392-025-02148-4, PMID: 39979279 PMC11842613

[170] Yi X Jin X Hu Y Shen Z Zheng X Luo D . Drug delivery systems for overcoming physical barriers in cancer therapy. Mol Pharmaceutics. (2025). doi: 10.1021/acs.molpharmaceut.5c00474, PMID: 41001833

[171] Pernot S Evrard S Khatib A-M . The give-and-take interaction between the tumor microenvironment and immune cells regulating tumor progression and repression. Front Immunol. (2022) 13:2022. doi: 10.3389/fimmu.2022.850856, PMID: 35493456 PMC9043524

[172] Imtiaz S Ferdous UT Nizela A Hasan A Shakoor A Zia AW . Mechanistic study of cancer drug delivery: Current techniques, limitations, and future prospects. Eur J Medicinal Chem. (2025) 290:117535. doi: 10.1016/j.ejmech.2025.117535, PMID: 40132495

[173] Singh AK Malviya R Prajapati B Singh S Yadav D Kumar A . Nanotechnology-aided advancement in combating the cancer metastasis. Pharmaceuticals. (2023) 16:899. doi: 10.3390/ph16060899, PMID: 37375846 PMC10304141

[174] Waheed I Ali A Tabassum H Khatoon N Lai W-F Zhou X . Lipid-based nanoparticles as drug delivery carriers for cancer therapy. Front Oncol. (2024) 14:2024. doi: 10.3389/fonc.2024.1296091, PMID: 38660132 PMC11040677

[175] Sahu T Ratre YK Chauhan S Bhaskar LVKS Nair MP Verma HK . Nanotechnology based drug delivery system: Current strategies and emerging therapeutic potential for medical science. J Drug Delivery Sci Technol. (2021) 63:102487. doi: 10.1016/j.jddst.2021.102487

[176] Manzari MT Shamay Y Kiguchi H Rosen N Scaltriti M Heller DA . Targeted drug delivery strategies for precision medicines. Nat Rev Mater. (2021) 6:351–70. doi: 10.1038/s41578-020-00269-6, PMID: 34950512 PMC8691416

[177] Raj S Khurana S Choudhari R Kesari KK Kamal MA Garg N . Specific targeting cancer cells with nanoparticles and drug delivery in cancer therapy. Semin Cancer Biol. (2021) 69:166–77. doi: 10.1016/j.semcancer.2019.11.002, PMID: 31715247

[178] Ciftci F Özarslan AC Kantarci İC Yelkenci A Tavukcuoglu O Ghorbanpour M . Advances in drug targeting, drug delivery, and nanotechnology applications: therapeutic significance in cancer treatment. Pharmaceutics. (2025) 17:121. doi: 10.3390/pharmaceutics17010121, PMID: 39861768 PMC11769154

[179] Moradi Kashkooli F Soltani M Souri M . Controlled anti-cancer drug release through advanced nano-drug delivery systems: Static and dynamic targeting strategies. J Control Release. (2020) 327:316–49. doi: 10.1016/j.jconrel.2020.08.012, PMID: 32800878

[180] Mahajan AS Stegh AH . Spherical nucleic acids as precision therapeutics for the treatment of cancer-from bench to bedside. Cancers (Basel). (2022) 14. doi: 10.3390/cancers14071615, PMID: 35406387 PMC8996871

[181] Keshavarz A Pourbagheri-Sigaroodi A Zafari P Bagheri N Ghaffari SH Bashash D . Toll-like receptors (TLRs) in cancer; with an extensive focus on TLR agonists and antagonists. IUBMB Life. (2021) 73:10–25. doi: 10.1002/iub.2412, PMID: 33217774

[182] Serras A Faustino C Pinheiro L . Functionalized polymeric micelles for targeted cancer therapy: steps from conceptualization to clinical trials. Pharmaceutics. (2024) 16:1047. doi: 10.3390/pharmaceutics16081047, PMID: 39204392 PMC11359152

[183] Sun L Liu H Ye Y Lei Y Islam R Tan S . Smart nanoparticles for cancer therapy. Signal Transduction Targeted Ther. (2023) 8:418. doi: 10.1038/s41392-023-01642-x, PMID: 37919282 PMC10622502

[184] Wang H Li Y Qi Y Zhao E Kong X Yang C . Pegylated liposomal doxorubicin, docetaxel, and trastuzumab as neoadjuvant treatment for HER2-positive breast cancer patients: A phase II and biomarker study. Front Oncol. (2022) 12:909426. doi: 10.3389/fonc.2022.909426, PMID: 35875123 PMC9304895

[185] Sun X Lin Y Zhong X Fan C Liu Z Chen X . Alendronate-functionalized polymeric micelles target icaritin to bone for mitigating osteoporosis in a rat model. J Control Release. (2024) 376:37–51. doi: 10.1016/j.jconrel.2024.10.002, PMID: 39368708

[186] Bahadur S Jha MK . Emerging nanoformulations for drug targeting to brain through intranasal delivery: A comprehensive review. J Drug Delivery Sci Technol. (2022) 78:103932. doi: 10.1016/j.jddst.2022.103932

[187] Fan D Cao Y Cao M Wang Y Cao Y Gong T . Nanomedicine in cancer therapy. Signal Transduction Targeted Ther. (2023) 8:293. doi: 10.1038/s41392-023-01536-y, PMID: 37544972 PMC10404590

[188] Biswas N Chakrabarti S . Artificial intelligence (AI)-based systems biology approaches in multi-omics data analysis of cancer. Front Oncol. (2020) 10:588221. doi: 10.3389/fonc.2020.588221, PMID: 33154949 PMC7591760

[189] Nicora G Vitali F Dagliati A Geifman N Bellazzi R . Integrated multi-omics analyses in oncology: A review of machine learning methods and tools. Front Oncol. (2020) 10:2020. doi: 10.3389/fonc.2020.01030, PMID: 32695678 PMC7338582

[190] Vlachavas EI Bohn J Ückert F Nürnberg S . A detailed catalogue of multi-omics methodologies for identification of putative biomarkers and causal molecular networks in translational cancer research. Int J Mol Sci. (2021) 22. doi: 10.3390/ijms22062822, PMID: 33802234 PMC8000236

[191] Marshall JL Peshkin BN Yoshino T Vowinckel J Danielsen HE Melino G . The essentials of multiomics. Oncologist. (2022) 27:272–84. doi: 10.1093/oncolo/oyab048, PMID: 35380712 PMC8982374

[192] Tran KA Kondrashova O Bradley A Williams ED Pearson JV Waddell N . Deep learning in cancer diagnosis, prognosis and treatment selection. Genome Med. (2021) 13:1–17. doi: 10.1186/s13073-021-00968-x, PMID: 34579788 PMC8477474

[193] Arjmand B Hamidpour SK Tayanloo-Beik A Goodarzi P Aghayan HR Adibi H . Machine learning: a new prospect in multi-omics data analysis of cancer. Front Genet. (2022) 13:824451. doi: 10.3389/fgene.2022.824451, PMID: 35154283 PMC8829119

[194] Mertins P Mani D Ruggles KV Gillette MA Clauser KR Wang P . Proteogenomics connects somatic mutations to signalling in breast cancer. Nature. (2016) 534:55–62. doi: 10.1038/nature18003, PMID: 27251275 PMC5102256

[195] Hasin Y Seldin M Lusis A . Multi-omics approaches to disease. Genome Biol. (2017) 18:1–15. doi: 10.1186/s13059-017-1215-1, PMID: 28476144 PMC5418815

[196] Vogelstein B Papadopoulos N Velculescu VE Zhou S Diaz LA Jr. Kinzler KW . Cancer genome landscapes. Science. (2013) 339:1546–58. doi: 10.1126/science.1235122, PMID: 23539594 PMC3749880

[197] Zhang H Liu T Zhang Z Payne SH Zhang B McDermott JE . Integrated proteogenomic characterization of human high-grade serous ovarian cancer. Cell. (2016) 166:755–65. doi: 10.1016/j.cell.2016.05.069, PMID: 27372738 PMC4967013

[198] Abbosh C Frankell AM Harrison T Kisistok J Garnett A Johnson L . Tracking early lung cancer metastatic dissemination in TRACERx using ctDNA. Nature. (2023) 616:553–62. doi: 10.1038/s41586-023-05776-4, PMID: 37055640 PMC7614605

[199] Martínez-Ruiz C Black JRM Puttick C Hill MS Demeulemeester J Larose Cadieux E . Genomic–transcriptomic evolution in lung cancer and metastasis. Nature. (2023) 616:543–52., PMID: 37046093 10.1038/s41586-023-05706-4 PMC10115639

[200] Janni W Rack B Friedl TWP Hartkopf AD Wiesmüller L Pfister K . Detection of minimal residual disease and prediction of recurrence in breast cancer using a plasma-only circulating tumor DNA assay. ESMO Open. (2025) 10:104296. doi: 10.1016/j.esmoop.2025.104296, PMID: 40120523 PMC11982450

[201] Gong J Hendifar A Gangi A Zaghiyan K Atkins K Nasseri Y . Clinical applications of minimal residual disease assessments by tumor-informed and tumor-uninformed circulating tumor DNA in colorectal cancer. Cancers (Basel). (2021) 13. doi: 10.3390/cancers13184547, PMID: 34572774 PMC8471730

[202] Li Y Dou Y Da Veiga Leprevost F Geffen Y Calinawan AP Aguet F . Proteogenomic data and resources for pan-cancer analysis. Cancer Cell. (2023) 41:1397–406. doi: 10.1016/j.ccell.2023.06.009, PMID: 37582339 PMC10506762

[203] Jaehnig EJ Fernandez-Martinez A Vashist TD Holt MV Williams L Lei JT . Proteogenomic analysis of the CALGB 40601 (Alliance) HER2+ breast cancer neoadjuvant trial reveals resistance biomarkers. Cell Rep Med. (2025) 6:102154. doi: 10.1016/j.xcrm.2025.102154, PMID: 40480221 PMC12208316

[204] Rodon J Soria JC Berger R Miller WH Rubin E Kugel A . Genomic and transcriptomic profiling expands precision cancer medicine: the WINTHER trial. Nat Med. (2019) 25:751–8. doi: 10.1038/s41591-019-0424-4, PMID: 31011205 PMC6599610

[205] Lim SB Lim CT Lim WT . Single-cell analysis of circulating tumor cells: why heterogeneity matters. Cancers (Basel). (2019) 11. doi: 10.3390/cancers11101595, PMID: 31635038 PMC6826423

[206] Williams MJ Vázquez-García I Tam G Wu M Varice N Havasov E . Tracking clonal evolution during treatment in ovarian cancer using cell-free DNA. Nature. (2025). doi: 10.1038/s41586-025-09580-0, PMID: 41034582 PMC12629990

[207] Leppä A-M Grimes K Jeong H Huang FY Andrades A Waclawiczek A . Single-cell multiomics analysis reveals dynamic clonal evolution and targetable phenotypes in acute myeloid leukemia with complex karyotype. Nat Genet. (2024) 56:2790–803. doi: 10.1038/s41588-024-01999-x, PMID: 39587361 PMC11631769

[208] Karlsson J Briggs M Vedi A Gisselsson D . Clonal evolution and therapy resistance in the era of precision cancer medicine: evolutionary trajectories in pediatric cancer. Semin Cancer Biol. (2025) 116:121–34. doi: 10.1016/j.semcancer.2025.10.001, PMID: 41067671

[209] Lawrence R Watters M Davies CR Pantel K Lu YJ . Circulating tumour cells for early detection of clinically relevant cancer. Nat Rev Clin Oncol. (2023) 20:487–500. doi: 10.1038/s41571-023-00781-y, PMID: 37268719 PMC10237083

[210] Wang X Bai L Kong L Guo Z . Advances in circulating tumor cells for early detection, prognosis and metastasis reduction in lung cancer. Front Oncol. (2024) 14:2024. doi: 10.3389/fonc.2024.1411731, PMID: 38974237 PMC11224453

[211] Ruan H Wang Z Sun Z Wei J Zhang L Ju H . Single-cell RNA sequencing reveals the characteristics of cerebrospinal fluid tumour environment in breast cancer and lung cancer leptomeningeal metastases. Clin Transl Med. (2022) 12:e885. doi: 10.1002/ctm2.885, PMID: 35678121 PMC9178395

[212] van Linder BMH Brandsma D Verhoeven E Jongejan L Monkhorst K de Langen AJ . Improving the diagnosis of leptomeningeal metastases by molecular profiling of cell-free DNA from cerebrospinal fluid. Eur J Cancer. (2025) 229:115783. doi: 10.1016/j.ejca.2025.115783, PMID: 40966899

[213] Kourou K Exarchos TP Exarchos KP Karamouzis MV Fotiadis DI . Machine learning applications in cancer prognosis and prediction. Comput Struct Biotechnol J. (2015) 13:8–17. doi: 10.1016/j.csbj.2014.11.005, PMID: 25750696 PMC4348437

[214] Albaradei S Thafar M Alsaedi A Van Neste C Gojobori T Essack M . Machine learning and deep learning methods that use omics data for metastasis prediction. Comput Struct Biotechnol J. (2021) 19:5008–18. doi: 10.1016/j.csbj.2021.09.001, PMID: 34589181 PMC8450182

[215] Merten C . A microfluidics platform for combinatorial drug screening on cancer biopsies. (2018)., PMID: 29934552 10.1038/s41467-018-04919-w PMC6015045

[216] Cova TF Bento DJ Nunes SC . Computational approaches in theranostics: mining and predicting cancer data. Pharmaceutics. (2019) 11:119. doi: 10.3390/pharmaceutics11030119, PMID: 30871264 PMC6471740

[217] Johnson D Osborne J Wang Z Marias K . Computer simulation, visualization, and image processing of cancer data and processes. London, England: SAGE Publications Sage UK (2015). 10.4137/CIN.S37982 PMC4711392 26798209

[218] Jung HD Sung YJ Kim HU . Omics and computational modeling approaches for the effective treatment of drug-resistant cancer cells. Front Genet. (2021) 12:742902. doi: 10.3389/fgene.2021.742902, PMID: 34691155 PMC8527086

[219] Jean-Quartier C Jeanquartier F Jurisica I Holzinger A . In silico cancer research towards 3R. BMC Cancer. (2018) 18:1–12. doi: 10.1186/s12885-018-4302-0, PMID: 29649981 PMC5897933

[220] Choi S Whitman MA Shimpi AA Sempertegui ND Chiou AE Druso JE . Bone-matrix mineralization dampens integrin-mediated mechanosignalling and metastatic progression in breast cancer. Nat Biomed Eng. (2023) 7:1455–72. doi: 10.1038/s41551-023-01077-3, PMID: 37550422

[221] Malandrino A Kamm RD Moeendarbary E . *In vitro* modeling of mechanics in cancer metastasis. ACS Biomaterials Sci Eng. (2018) 4:294–301. doi: 10.1021/acsbiomaterials.7b00041, PMID: 29457129 PMC5811931

[222] Zhang S Liu K Liu Y Hu X Gu X . The role and application of bioinformatics techniques and tools in drug discovery. Front Pharmacol. (2025) 16:1547131. doi: 10.3389/fphar.2025.1547131, PMID: 40017606 PMC11865229

[223] Altman R . Translational bioinformatics: linking the molecular world to the clinical world. Clin Pharmacol Ther. (2012) 91:994–1000. doi: 10.1038/clpt.2012.49, PMID: 22549287 PMC4154360

[224] Lu D Lu T Chen X Chen E Ding J Xu B . Cancer bioinformatics, its impacts on cancer therapy. Metabolomics. (2015) 5:e133.

[225] Afzal M Islam SR Hussain M Lee S . Precision medicine informatics: principles, prospects, and challenges. IEEE Access. (2020) 8:13593–612. doi: 10.1109/ACCESS.2020.2965955

[226] Ansori AN Antonius Y Susilo RJ Hayaza S Kharisma VD Parikesit AA . Application of CRISPR-Cas9 genome editing technology in various fields: A review. Narra J. (2023) 3:e184. doi: 10.52225/narra.v3i2.184, PMID: 38450259 PMC10916045

[227] Martinez-Lage M Torres-Ruiz R Rodriguez-Perales S . CRISPR/Cas9 technology: applications and human disease modeling. Prog Mol Biol Trans Sci. (2017) 152:23–48. doi: 10.1016/bs.pmbts.2017.09.002, PMID: 29150003

[228] Doudna JA Charpentier E . The new frontier of genome engineering with CRISPR-Cas9. Science. (2014) 346:1258096. doi: 10.1126/science.1258096, PMID: 25430774

[229] Sander JD Joung JK . CRISPR-Cas systems for editing, regulating and targeting genomes. Nat Biotechnol. (2014) 32:347–55. doi: 10.1038/nbt.2842, PMID: 24584096 PMC4022601

[230] Turetta M Ben FD Brisotto G Biscontin E Bulfoni M Cesselli D . Emerging technologies for cancer research: towards personalized medicine with microfluidic platforms and 3D tumor models. Curr Med Chem. (2018) 25:4616–37. doi: 10.2174/0929867325666180605122633, PMID: 29874987 PMC6302350

[231] Zakari S Niels NK Olagunju GV Nnaji PC Ogunniyi O Tebamifor M . Emerging biomarkers for non-invasive diagnosis and treatment of cancer: a systematic review. Front Oncol. (2024) 14:1405267. doi: 10.3389/fonc.2024.1405267, PMID: 39132504 PMC11313249

[232] Eibl RH Schneemann M . Liquid biopsy and glioblastoma. Explor Target Antitumor Ther. (2023) 4:28–41. doi: 10.37349/etat.2023.00121, PMID: 36937320 PMC10017188

[233] Sorrells S McKinnon KE McBratney A Sumey C . Longitudinal and multi-tissue molecular diagnostics track somatic BRCA2 reversion mutations that correct the open reading frame of germline alteration upon clinical relapse. NPJ Genom Med. (2021) 6:17. doi: 10.1038/s41525-021-00181-0, PMID: 33619265 PMC7900170

[234] Stanta G Bonin S . A practical approach to tumor heterogeneity in clinical research and diagnostics. Pathobiology. (2018) 85:7–17. doi: 10.1159/000477813, PMID: 28750401

[235] Li L Zheng X Zhou Q Villanueva N Nian W Liu X . Metabolomics-based discovery of molecular signatures for triple negative breast cancer in asian female population. Sci Rep. (2020) 10:370. doi: 10.1038/s41598-019-57068-5, PMID: 31941951 PMC6962155

[236] Mathur L Ballinger M Utharala R Merten CA . Microfluidics as an enabling technology for personalized cancer therapy. Small. (2020) 16:e1904321. doi: 10.1002/smll.201904321, PMID: 31747127

[237] Alizadeh AA Aranda V Bardelli A Blanpain C Bock C Borowski C . Toward understanding and exploiting tumor heterogeneity. Nat Med. (2015) 21:846–53. doi: 10.1038/nm.3915, PMID: 26248267 PMC4785013

[238] Danese E Lippi G Montagnana M . Epigenetica e cancro del colon-retto: limiti e prospettive. La Rivista Italiana della Med di Laboratorio - Ital J Lab Med. (2018) 14:8–10. doi: 10.1007/s13631-018-0175-0

[239] Malone ER Oliva M Sabatini PJB Stockley TL Siu LL . Molecular profiling for precision cancer therapies. Genome Med. (2020) 12:8. doi: 10.1186/s13073-019-0703-1, PMID: 31937368 PMC6961404

[240] Clarke R Kraikivski P Jones BC Sevigny CM Sengupta S Wang Y . A systems biology approach to discovering pathway signaling dysregulation in metastasis. Cancer Metastasis Rev. (2020) 39:903–18. doi: 10.1007/s10555-020-09921-7, PMID: 32776157 PMC7487029

[241] Suhail Y Cain MP Vanaja K Kurywchak PA Levchenko A Kalluri R . Systems biology of cancer metastasis. Cell Syst. (2019) 9:109–27. doi: 10.1016/j.cels.2019.07.003, PMID: 31465728 PMC6716621

[242] Teschendorff AE Severini S . Increased entropy of signal transduction in the cancer metastasis phenotype. BMC Syst Biol. (2010) 4:1–15. doi: 10.1186/1752-0509-4-104, PMID: 20673354 PMC2925356

[243] Papadas A Deb G Cicala A Officer A Hope C Pagenkopf A . Stromal remodeling regulates dendritic cell abundance and activity in the tumor microenvironment. Cell Rep. (2022) 40. doi: 10.1016/j.celrep.2022.111201, PMID: 35977482 PMC9402878

[244] Schild T Low V Blenis J Gomes AP . Unique metabolic adaptations dictate distal organ-specific metastatic colonization. Cancer Cell. (2018) 33:347–54. doi: 10.1016/j.ccell.2018.02.001, PMID: 29533780 PMC5889305

[245] Wang C Luo D . The metabolic adaptation mechanism of metastatic organotropism. Exp Hematol Oncol. (2021) 10:30. doi: 10.1186/s40164-021-00223-4, PMID: 33926551 PMC8082854

[246] Cheng M Sun Q Duan H Chen C . Global hotspots and trends in pre-metastatic niche research: a bibliometric analysis (2005-2024). Front Immunol. (2025) 16:1552053. doi: 10.3389/fimmu.2025.1552053, PMID: 40510361 PMC12159010

[247] Ragusa M Barbagallo C Brex D Caponnetto A Cirnigliaro M Battaglia R . Molecular crosstalking among noncoding RNAs: a new network layer of genome regulation in cancer. Int J Genomics. (2017) 2017:4723193. doi: 10.1155/2017/4723193, PMID: 29147648 PMC5632862

[248] Anand U Dey A Chandel AKS Sanyal R Mishra A Pandey DK . Cancer chemotherapy and beyond: Current status, drug candidates, associated risks and progress in targeted therapeutics. Genes Dis. (2023) 10:1367–401. doi: 10.1016/j.gendis.2022.02.007, PMID: 37397557 PMC10310991

[249] Izadi N Solár P Hašanová K Zamani A Akbar MS Mrázová K . Breaking boundaries: role of the brain barriers in metastatic process. Fluids Barriers CNS. (2025) 22, 3. doi: 10.1186/s12987-025-00618-z, PMID: 39780275 PMC11708195

[250] Lei ZN Tian Q Teng QX Wurpel JN Zeng L Pan Y . Understanding and targeting resistance mechanisms in cancer. MedComm. (2023) 4:e265. 37229486 10.1002/mco2.265 PMC10203373

[251] Joyce JA Pollard JW . Microenvironmental regulation of metastasis. Nat Rev Cancer. (2009) 9:239–52. 10.1038/nrc2618 PMC3251309 19279573

[252] Ruan L Wang L . Adoptive cell therapy against tumor immune evasion: mechanisms, innovations, and future directions. Front Oncol. (2025) 15:1530541., PMID: 40094019 10.3389/fonc.2025.1530541 PMC11906336

[253] Passaro A Al Bakir M Hamilton EG Diehn M André F Roy-Chowdhuri S . Cancer biomarkers: emerging trends and clinical implications for personalized treatment. Cell. (2024) 187:1617–35., PMID: 38552610 10.1016/j.cell.2024.02.041 PMC7616034

[254] Sun D Gao W Hu H Zhou S . Why 90% of clinical drug development fails and how to improve it? Acta Pharm Sin B. (2022) 12:3049–62. 10.1016/j.apsb.2022.02.002 PMC9293739 35865092

[255] Hughes VS Siemann DW . Failures in preclinical and clinical trials of c-Met inhibitors: evaluation of pathway activity as a promising selection criterion. Oncotarget. (2019) 10:184–97. doi: 10.18632/oncotarget.26546, PMID: 30719213 PMC6349429

[256] Anderson RL Balasas T Callaghan J Coombes RC Evans J Hall JA . A framework for the development of effective anti-metastatic agents. Nat Rev Clin Oncol. (2019) 16:185–204. doi: 10.1038/s41571-018-0134-8, PMID: 30514977 PMC7136167

[257] Manzari-Tavakoli A Babajani A Tavakoli MM Safaeinejad F Jafari A . Integrating natural compounds and nanoparticle-based drug delivery systems: A novel strategy for enhanced efficacy and selectivity in cancer therapy. Cancer Med. (2024) 13:e7010., PMID: 38491817 10.1002/cam4.7010 PMC10943377

[258] Venturini J Chakraborty A Baysal MA Tsimberidou AM . Developments in nanotechnology approaches for the treatment of solid tumors. Exp Hematol Oncol. (2025) 14:1–47. doi: 10.1186/s40164-025-00656-1, PMID: 40390104 PMC12090476

[259] Sabir S Thani ASB Abbas Q . Nanotechnology in cancer treatment: revolutionizing strategies against drug resistance. Front Bioengineering Biotechnol. (2025) 13:1548588. doi: 10.3389/fbioe.2025.1548588, PMID: 40370595 PMC12075138

[260] Kong X Zhang J Chen S Wang X Xi Q Shen H . Immune checkpoint inhibitors: breakthroughs in cancer treatment. Cancer Biol Med. (2024) 21:451–72. doi: 10.20892/j.issn.2095-3941.2024.0055, PMID: 38801082 PMC11208906

[261] Gonciar D Mocan T Matea CT Zdrehus C Mosteanu O Mocan L . Nanotechnology in metastatic cancer treatment: Current Achievements and Future Research Trends. J Cancer. (2019) 10:1358–69. doi: 10.7150/jca.28394, PMID: 31031845 PMC6485233

[262] Xia Y Duan S Han C Jing C Xiao Z Li C . Hypoxia-responsive nanomaterials for tumor imaging and therapy. Front Oncol. (2022) 12:1089446. doi: 10.3389/fonc.2022.1089446, PMID: 36591450 PMC9798000

[263] Avula LR Grodzinski P . Nanotechnology-aided advancement in the combating of cancer metastasis. Cancer Metastasis Rev. (2022) 41:383–404. doi: 10.1007/s10555-022-10025-7, PMID: 35366154 PMC8975728

[264] Zhang P Meng J Li Y Yang C Hou Y Tang W . Nanotechnology-enhanced immunotherapy for metastatic cancer. Innovation. (2021) 2:100174., PMID: 34766099 10.1016/j.xinn.2021.100174 PMC8571799

[265] Zhao Y Zheng X Zheng Y Chen Y Fei W Wang F . Extracellular matrix: emerging roles and potential therapeutic targets for breast cancer. Front Oncol. (2021) 11:2021. doi: 10.3389/fonc.2021.650453, PMID: 33968752 PMC8100244

[266] Cao L Zhu Y Wang W Wang G Zhang S Cheng H . Emerging nano-based strategies against drug resistance in tumor chemotherapy. Front Bioengineering Biotechnol. (2021) 9:2021. doi: 10.3389/fbioe.2021.798882, PMID: 34950650 PMC8688801

[267] Ghassemifar V Zahedi A Meigoli MSS Mokhtari T Zadeh SSM Parsafar M . Harnessing nanotechnology to modulate hypoxic tumor microenvironments: Enhanced strategies for oncological innovations. Biomedicine Pharmacotherapy. (2025) 191:118543. doi: 10.1016/j.biopha.2025.118543, PMID: 40939258

[268] Kopac T . Leveraging artificial intelligence and machine learning for characterizing protein corona, nanobiological interactions, and advancing drug discovery. Bioengineering. (2025) 12:312. doi: 10.3390/bioengineering12030312, PMID: 40150776 PMC11939375

